# Nurse-led Telehealth Intervention for Rehabilitation (Telerehabilitation) Among Community-Dwelling Patients With Chronic Diseases: Systematic Review and Meta-analysis

**DOI:** 10.2196/40364

**Published:** 2022-11-02

**Authors:** Athena Yin Lam Lee, Arkers Kwan Ching Wong, Tommy Tsz Man Hung, Jing Yan, Shulan Yang

**Affiliations:** 1 School of Nursing The Hong Kong Polytechnic University Hung Hom Hong Kong; 2 Zhejiang Hospital Zhejiang China

**Keywords:** chronic disease, telerehabilitation, meta-analysis, telemedicine, nurses, outpatients

## Abstract

**Background:**

Chronic diseases are putting huge pressure on health care systems. Nurses are widely recognized as one of the competent health care providers who offer comprehensive care to patients during rehabilitation after hospitalization. In recent years, telerehabilitation has opened a new pathway for nurses to manage chronic diseases at a distance; however, it remains unclear which chronic disease patients benefit the most from this innovative delivery mode.

**Objective:**

This study aims to summarize current components of community-based, nurse-led telerehabilitation programs using the chronic care model; evaluate the effectiveness of nurse-led telerehabilitation programs compared with traditional face-to-face rehabilitation programs; and compare the effects of telerehabilitation on patients with different chronic diseases.

**Methods:**

A systematic review and meta-analysis were performed using 6 databases for articles published from 2015 to 2021. Studies comparing the effectiveness of telehealth rehabilitation with face-to-face rehabilitation for people with hypertension, cardiac diseases, chronic respiratory diseases, diabetes, cancer, or stroke were included. Quality of life was the primary outcome. Secondary outcomes included physical indicators, self-care, psychological impacts, and health-resource use. The revised Cochrane risk of bias tool for randomized trials was employed to assess the methodological quality of the included studies. A meta-analysis was conducted using a random-effects model and illustrated with forest plots.

**Results:**

A total of 26 studies were included in the meta-analysis. Telephone follow-ups were the most commonly used telerehabilitation delivery approach. Chronic care model components, such as nurses-patient communication, self-management support, and regular follow-up, were involved in all telerehabilitation programs. Compared with traditional face-to-face rehabilitation groups, statistically significant improvements in quality of life (cardiac diseases: standard mean difference [SMD] 0.45; 95% CI 0.09 to 0.81; *P*=.01; heterogeneity: *X*^2^_1_=1.9; *I*^2^=48%; *P*=.16; chronic respiratory diseases: SMD 0.18; 95% CI 0.05 to 0.31; *P*=.007; heterogeneity: *X*^2^_2_=1.7; *I*^2^=0%; *P*=.43) and self-care (cardiac diseases: MD 5.49; 95% CI 2.95 to 8.03; *P*<.001; heterogeneity: *X*^2^_5_=6.5; *I*^2^=23%; *P*=.26; diabetes: SMD 1.20; 95% CI 0.55 to 1.84; *P*<.001; heterogeneity: *X*^2^_4_=46.3; *I*^2^=91%; *P*<.001) were observed in the groups that used telerehabilitation. For patients with any of the 6 targeted chronic diseases, those with hypertension and diabetes experienced significant improvements in their blood pressure (systolic blood pressure: MD 10.48; 95% CI 2.68 to 18.28; *P*=.008; heterogeneity: *X*^2^_1_=2.2; I^2^=54%; *P*=0.14; diastolic blood pressure: MD 1.52; 95% CI –10.08 to 13.11, *P*=.80; heterogeneity: *X*^2^1=11.5; I^2^=91%; *P*<.001), and hemoglobin A1c (MD 0.19; 95% CI –0.19 to 0.57 *P*=.32; heterogeneity: *X*^2^_4_=12.4; I^2^=68%; *P*=.01) levels. Despite these positive findings, telerehabilitation was found to have no statistically significant effect on improving patients’ anxiety level, depression level, or hospital admission rate.

**Conclusions:**

This review showed that telerehabilitation programs could be beneficial to patients with chronic disease in the community. However, better designed nurse-led telerehabilitation programs are needed, such as those involving the transfer of nurse-patient clinical data. The heterogeneity between studies was moderate to high. Future research could integrate the chronic care model with telerehabilitation to maximize its benefits for community-dwelling patients with chronic diseases.

**Trial Registration:**

International Prospective Register of Systematic Reviews CRD42022324676; https://www.crd.york.ac.uk/prospero/display_record.php?RecordID=324676

## Introduction

“Telehealth” refers to the delivery of health services through technology when health care providers and patients are separated by distance [1]. One of the branches of telehealth, telerehabilitation, is defined as the use of a telehealth approach to provide rehabilitation care to people with long-term chronic diseases [2]. Telerehabilitation programs employ communication and information technology, such as telephones and videoconferencing, as a delivery channel to provide not only exercise training, but also self-management education and health behavior modifications to patients with chronic disease who are not receiving hospital care [3,4]. Despite offering convenience, telerehabilitation also has well-known disadvantages, such as technical issues, limitations on carrying out procedures that require physical contact, and security breaches.

Many systematic reviews have been published in recent years on the effectiveness of telerehabilitation programs for those with one specific chronic disease (eg, cardiac diseases, respiratory diseases, stroke, or neurological diseases). A previous systematic review suggested that there is controversy over the effectiveness of telerehabilitation and that its impacts could differ depending on which chronic disease a person has [5]. However, to our knowledge, no reviews have been published on which chronic disease patients would benefit most from telerehabilitation programs. The aim in this present review is to address these research and service gaps by comparing the effects of telerehabilitation programs on people with different chronic diseases. If proven successful, the findings can aid the government and policymakers in better allocating health care resources, foster the development of telerehabilitation programs during and beyond the COVID-19 pandemic, and improve the quality of community care services.

The objectives of the review are to identify the intervention components of current nurse-led telerehabilitation programs for community-dwelling people with chronic diseases, to evaluate the effectiveness of nurse-led telerehabilitation programs compared with traditional face-to-face rehabilitation programs, and to compare the effects of telerehabilitation on patients with different chronic diseases.

## Methods

### Overview

A systematic review and meta-analysis was conducted following the PRISMA (Preferred Reporting Items for Systematic Reviews and Meta-Analyses) 2020 statement [6]. This study was registered in the International Prospective Register of Systematic Reviews (PROSPERO; CRD42022324676).

### Literature Search

The literature search was performed by 2 independent reviewers (AYLL and AKCW) without the involvement of librarians. PubMed, MEDLINE, CINAHL, Embase, PsycInfo, and the Cochrane Central Register of Controlled Trials were searched for articles published from 2015 to 2021, with the aim of capturing the most updated telerehabilitation approaches under rapid technological development. Handsearching was performed using Google Scholar and the reference lists of included papers. Gray literature, such as abstracts and editorials, were excluded as most of these articles are not peer-reviewed and their inclusion would have lowered the quality of evidence. Search strategies for all databases were constructed based on the key search terms, which included “telerehabilitation,” “chronic disease,” “nursing,” “multi-disciplinary,” and “randomized controlled trial.” The search was further expanded by the inclusion of different chronic diseases and medical subject headings mesh terms (Multimedia Appendix 1).

### Eligibility Criteria

#### Overview

Studies that included people with hypertension, cardiac diseases (coronary artery diseases, heart failure), chronic respiratory diseases (asthma, chronic obstructive pulmonary disease [COPD]), diabetes, cancer, or stroke were the target of this review because these are common diseases among people in the community that require the provision of long-term nursing rehabilitation care. Articles were screened using the following eligibility criteria constructed using the patient, intervention, comparison, and outcome (PICO) strategy.

#### Inclusion Criteria

The inclusion criteria for articles were the following: participants aged 18 years or above, diagnosed with one of the targeted chronic diseases, and living independently in the community outside health care facilities; telerehabilitation employed as the intervention delivery channel in 1 arm of the intervention (the channel could include telephone calls, smartphone apps, videoconferencing, or SMS text messaging), with nurses providing of at least 50% of the program in terms of the frequency or duration of the provision of care; comparison to conventional face-to-face center-based consultations or a waitlist control; outcomes of quality of life, disease-specific physical indicators, self-care ability, psychological outcomes (depression, anxiety), and health-resource use; and a randomized controlled trial study design.

#### Exclusion Criteria

The exclusion criteria for articles were the following: participants were patients living in assisted residential care facilities (ie, a nursing home) and interventions were telerehabilitation programs conducted in hospital settings where the purpose of the program was to provide education or training only to health care professionals.

### Study Selection

**Figure 1:**
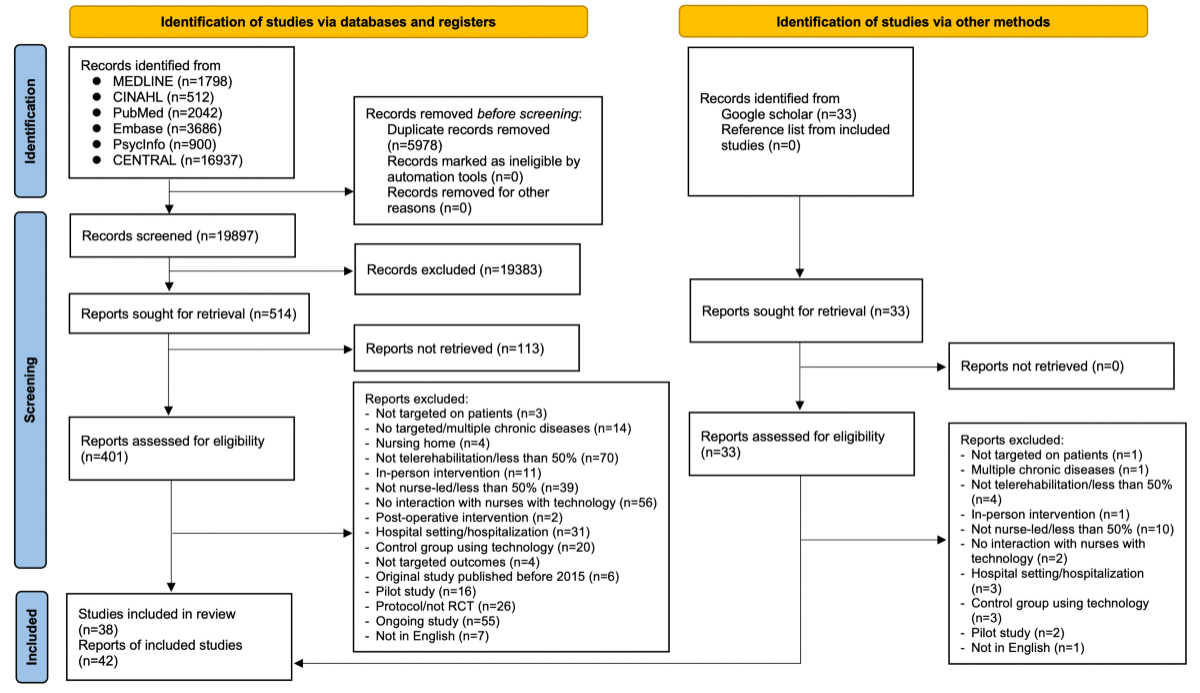
PRISMA (Preferred Reporting Items for Systematic Reviews and Meta-Analyses) 2020 flowchart (adapted from Page et al [6], which is published under Creative Commons Attribution 4.0 International License [7]). CENTRAL: The Cochrane Central Register of Controlled Trials; RCT: randomized controlled trial.

The literature screening process is reported using the PRISMA flowchart (Figure 1). The search results were retrieved and imported into EndNote X9 (Clarivate) for the removal of duplicates after the literature search. Articles were screened by title and abstract, which was followed by an examination of the full text by 2 reviewers (AYLL and AKCW) working independently. For the handsearching, the same 2 reviewers independently screened the full text of articles. Any disagreements among the reviewers were resolved through discussion.

### Data Extraction

The following variables were extracted and are listed in Multimedia Appendix 1: author, year of publication, study characteristics (study location, study population), intervention characteristics (providers, study duration, intervention group, control group), data collection timepoint, outcome variables, outcome measures, and results.

The interventions of all included studies were extracted according to the chronic care model. The six components of the model are as follows: (1) active, two-way interactions between an informed patient and proactive health care providers; (2) effective self-management support during communication; (3) an intact delivery system design with regular follow-ups for evaluation; (4) proper decision support from expertise, protocols, or training; (5) a clinical information system between patients and health care providers for managing the patients’ clinical data; and (6) community resources [8].

### Quality Assessment

The Cochrane risk of bias tool was used to identify the potential risk of bias in the included studies [9]. Two reviewers (AYLL and AKCW) performed the quality appraisal independently and resolved any disagreements through discussion.

### Statistical Analysis

A meta-analysis was conducted using Review Manager version 5.4.1 (The Cochrane Collaboration) and illustrated using a forest plot when at least 2 studies were measured for the same outcomes for a chronic disease at the longest follow-up timepoint [10]. Under the random-effects model, a mean difference (MD; using the same measurement tool) or a standardized MD (SMD; using different measurement tools) and a 95% CI were calculated for continuous variables, while odds ratios (ORs) and the 95% CIs were computed using the Mantel-Haenszel method for dichotomous variables. The heterogeneity and significance of the results were assessed using a chi-squared test, *I*^2^ statistics, and a *P* value (<.05). The value of *I*^2^ could be interpreted as indicating unimportant (0%-40%), moderate (30%-60%), substantial (50%-90%), or considerable (75%-100%) heterogeneity [10]. Publication bias was checked by visualization of a funnel plot [10].

## Results

### Screening Process

The screening process is reported in Figure 1. A total of 434 papers were included for a full-text screening, and eventually, 38 studies met the criteria for inclusion in this review, 26 of which had data available for a meta-analysis. The characteristics of all of the included studies are presented in the data extraction table (Multimedia Appendix 2).

### Study Characteristics

A total of 9677 participants with a mean age of 63.75 years were included in the 38 studies. Of these, 5105 received telerehabilitation and 4572 received conventional face-to-face consultations; 6 studies were 3-armed randomized controlled trials [11-16], and the remaining studies were 2-armed studies. Among the 38 included studies, 9 targeted patients with cardiac disease [15-23], 9 targeted chronic patients with respiratory disease [24-32], 9 targeted patients with diabetes [11,12,33-39], 4 targeted patients with hypertension [14,40-42], 4 targeted patients with cancer [43-46], and 3 targeted patients with stroke [13,47,48].

### Intervention Characteristics

The programs were performed by registered nurses (n=20), specialty nurses (n=8), advanced practice nurses (n=3), community nurses (n=1), a nurse case manager (n=1), or people involved in more than one health-related discipline (n=5). The study periods ranged from 4 weeks to 36 months, with 8 weeks (n=8), 12 weeks (n=7), and 24 weeks (n=7) being the most common durations.

The technologies used in telerehabilitation programs are summarized in Table 1. Generally, the nurse telephone follow-up (n=26) was the most commonly adopted nurse-led telerehabilitation delivery channel for all chronic disease patients, followed by telemonitoring (n=9) and videoconferencing (n=4). In addition, most of the studies involved nontechnological components in addition to telerehabilitation, such as distributing written educational materials (n=20), attending an in-person educational session (n=16), regular face-to-face training (n=10), and home visits by nurses (n=3). For the control groups, regular nursing consultations (n=30), paper-based educational materials (n=14), and an in-person educational session (n=6) were used.

**Table 1:** Telerehabilitation interventions for different chronic diseases.

	Hypertension, n	Cardiac diseases, n	Chronic respiratory diseases, n	Diabetes, n	Cancer, n	Stroke, n
Nurse follow-ups by telephone/video	3	6	5	8	2	3
Nurse follow-ups by SMS texts	2	0	0	0	0	0
Telemonitoring	0	3	4	1	0	0
Smartphone apps	0	0	0	2	2	0
Website	0	1	0	0	0	0
Exercise training	0	1	0	0	0	0

### Nurse Follow-Ups

A total of 32 studies conducted telerehabilitation programs through telephone (n=26) [11-13,15,17,19-22,24-28,33,35,​^37^-^43^,^46^-^48^], videoconferencing (n=4) [21,30,32,34], SMS text messaging (n=2) [14,41], or WhatsApp (n=1) [45] (Multimedia Appendix 3). Nurse-led counseling was mostly implemented weekly (n=4), monthly (n=5), or a combination of both (n=7). Seven studies did not report the frequency of their interventions. The contents of nursing follow-up included providing education on disease-specific knowledge (eg, COPD exacerbation, hypoglycemia) and self-care behavior (eg, medication adherence, lifestyle modification; n=14), addressing patients’ enquiries on disease self-management (n=8), monitoring patients’ signs and symptoms (n=6), conducting motivational interviewing (n=5), performing medication titration with collaboration of physicians (n=4), empowering goal-setting and personal plan implementation (n=4), and providing psychological support (n=3).

There were 2 studies framed by problem-solving theory, which supported chronic disease rehabilitation by developing behavioral plans and providing positive reinforcement during nurse-led phone counseling [13,28]. Another 2 studies used noninteractive information SMS texts and/or interactive SMS texts with nurses to provide education on chronic disease management and support on disease monitoring [14,41].

### Telemonitoring

Nine studies integrated telemonitoring in their telerehabilitation program for patients with heart failure, asthma, COPD, or diabetes [16,18,21,29-32,34,35] (Multimedia Appendix 4). Patients were instructed to measure their disease-specific physical indicators (eg, blood pressure, spirometry, oxygen saturation, respiratory rate, blood glucose level) and record their signs and symptoms daily (n=5), weekly (n=1), or from daily to weekly after the first few weeks of interventions (n=3). The data were transmitted to a shared platform by manual recording in tablet and mobile apps (n=3), auto-transmission from measurement tools to tablet (n=2), or SMS texts (n=1). In 7 studies, alerts were sent automatically to nurses if abnormal data were detected by decision-support systems. These decision-support systems were constructed according to research protocol (n=5) or through shared decision-making with patients (n=2). After receiving the alerts, the nurses would support these patients through continuing telephone follow-up (n=6), videoconferencing (n=2), or referring to physicians (n=1).

### Other Telerehabilitation Interventions

Apart from nurse follow-ups and telemonitoring, there were 4 studies that used smartphone apps to support chronic disease rehabilitation in the community [23,35,44,45]. The functions of these apps generally included provision of multimedia educational materials, monitoring of health behaviors, psychological support, chat functions, and discussion forums for nurse counseling. Two studies designed a website to provide education information on cardiac self-management [16] and monitor patients’ health status with the use of online health questionnaires [46]. In addition, one study provided online exercise training for patients with health failure through videoconferencing [23].

### Chronic Care Model

The overall chronic care model elements among all included studies are illustrated in Multimedia Appendix 5. The interventions in all of the studies are shown as being aligned with at least a part of the model. In all of the 38 included studies, regular interactions between patients and nurses, self-management support, and regular follow-ups were provided. In a total of 26 studies, decision support for nurses was provided through preintervention training, guidelines, or protocols. A clinical information system was involved in 10 studies with a telemonitoring component. There were 8 studies in which referrals were provided to available community resources.

### Quantitative Synthesis

**Figure 2:**
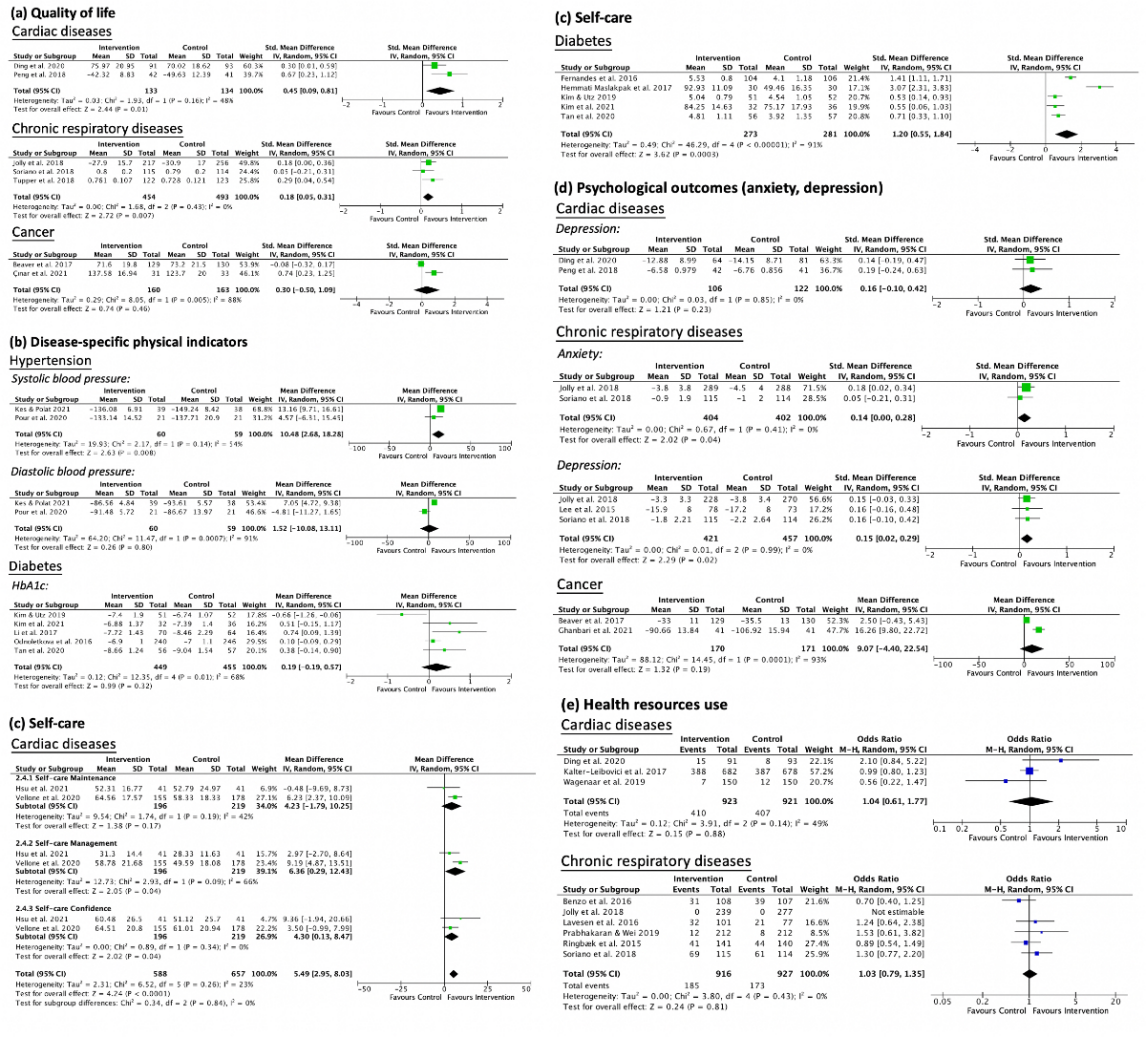
Forest plot on the effectiveness of community-based nurse-led telerehabilitation programs on (a) quality of life, (b) disease-specific physical indicators, (c) self-care ability, (d) psychological outcomes, and (e) health-resource use. std: standard.

Forest plots for all outcomes are shown in Figure 2.

### Quality of Life

#### Hypertension

One study reported that telerehabilitation programs had a positive effect on the quality of life of people with hypertension [40].

#### Cardiac Diseases

A significant improvement in the quality of life of cardiac disease patients who had received telerehabilitation was observed when compared with those who had received a nontechnological intervention (SMD 0.45; 95% CI 0.09-0.81; *P*=.01), with moderate heterogeneity (*X*^2^_1_=1.9; *I*^2^=48%; *P*=.16).

#### Chronic Respiratory Diseases

Pooled analyses of 3 studies showed that patients with COPD who received telerehabilitation had a significantly higher quality of life than did those who received conventional face-to-face rehabilitation (SMD 0.18; 95% CI 0.05-0.31; *P*=.007; heterogeneity: *X*^2^_2_=1.7; *I*^2^=0%; *P*=.43).

#### Diabetes (Type II)

For diabetes, 1 study [34] showed no significant difference, while 2 studies [38,39] reported an improvement in the quality of life of patients after receiving nurse-led telerehabilitation.

#### Cancer

From a meta-analysis of 2 studies, it was revealed that there was no significant difference in quality of life between the intervention and control groups of patients with cancer (SMD 0.30; 95% CI –0.50 to 1.09; heterogeneity: *P*=.46; *X*^2^_1_=8.1; *I*^2^=88%; *P*=.005).

### Disease-Specific Physical Indicators

#### Hypertension

Pooled intervention effects from 2 studies showed a significant improvement in the systolic blood pressure of patients through telerehabilitation (MD 10.48; 95% CI 2.68 to 18.28; *P*=.008), with moderate heterogeneity (*X*^2^_1_=2.8; *I*^2^=54%; *P*=.14). However, no significant difference was observed in their diastolic blood pressure (MD 1.52; 95% CI –10.08 to 13.11; *P*=.80; heterogeneity: *X*^2^_1_=11.5; *I*^2^=91%; *P*<.001).

#### Cardiac Diseases

Among those with heart failure, no significant differences between the telerehabilitation and control groups were observed in physical symptoms [17,21,23].

#### Chronic Respiratory Diseases

One included study reported that telerehabilitation had no effect on reducing the number of instances of COPD exacerbation or COPD symptom levels [31]. Another study also found no significant difference in dyspneic levels between those who received telerehabilitation and those who received conventional in-person follow-ups [26].

#### Diabetes (Type II)

A meta-analysis of 5 studies found that telerehabilitation had no significant effect on improving the hemoglobin A1c levels of patients (MD 0.19; 95% CI –0.19 to 0.57; *P*=.32). However, the above result might have been affected by an outlier since the findings showed substantial heterogeneity (*X*^2^_4_=12.4; *I*^2^=68%; *P*=.01).

#### Stroke

One study showed improved systolic blood pressure, diastolic blood pressure, and low-density lipoprotein levels in stroke survivors who had received a telerehabilitation program [47].

### Self-Care Ability

#### Hypertension

Only 1 study assessed the effect of telerehabilitation on self-care among patients with hypertension, and in that study, no significant difference was found between the groups [42].

#### Cardiac Diseases

A pooled analysis indicated that telerehabilitation could have a beneficial effect on the self-care ability of patients with cardiac diseases (MD 5.49; 95% CI 2.95 to 8.03; *P*<.001), with mild heterogeneity (*X*^2^_5_=6.5; *I*^2^=23%; *P*=.26). A subgroup analysis showed that participation in telerehabilitation led to a significant improvement in the participants’ self-care management (MD 6.36; 95% CI 0.29 to 12.43; *P*=.04) and self-care confidence (MD 4.30; 95% CI 0.13 to 8.47; *P*=.04) but not in their self-care maintenance (MD 4.23; 95% CI –1.79 to 10.25; *P*=.17).

#### Chronic Respiratory Diseases

One included study revealed that patients demonstrated a significant improvement in disease self-management after receiving telerehabilitation [27]. Nevertheless, another study showed no significant difference in self-management health behaviors between the telerehabilitation and onsite out-patient follow-up groups [25].

#### Diabetes (Type II)

The pooled SMD indicated that telerehabilitation had a significant positive effect on enhancing the self-care behavior of patients with diabetes when compared with conventional face-to-face nursing consultations (SMD 1.20; 95% CI 0.55-0.84; *P*<.001; heterogeneity: *X*^2^_4_=46.3; *I*^2^=91%; *P*<.001).

### Psychological Outcomes (Depression, Anxiety)

#### Cardiac Diseases

A meta-analysis showed that telerehabilitation had no significant effect on reducing the depression levels of patients who experience heart failure (SMD 0.16; 95% CI –0.10 to 0.42; *P*=.23; heterogeneity: *X*^2^_1_=0.03; *I*^2^=0%; *P*=.85).

#### Chronic Respiratory Diseases

Pooled analyses in 3 studies found there to be a significant reduction in anxiety (SMD 0.14; 95% CI 0.00-0.28; *P*=.04) and depression levels (SMD 0.15; 95% CI 0.02-0.29; *P*=.02) in patients with COPD.

#### Diabetes (Type II)

The only study that evaluated the effects of a telerehabilitation program on patients with diabetes showed an improvement in depression [36].

#### Cancer

The pooled MD showed no significant effect between groups on relieving anxiety (MD 9.07; 95% CI –4.40 to 22.54; *P*=.19; heterogeneity: *X*^2^_1_=14.5; *I*^2^=93%; *P*<.001).

#### Stroke

One study reported no significant differences in depression levels between stroke survivors who received telerehabilitation and those who received conventional face-to-face nurse consultations [13].

### Health-Resource Use

#### Cardiac Diseases

A pooled intervention effect of 3 studies showed that telerehabilitation had no significant effect on reducing hospitalizations of patients with heart failure (OR=1.04, 95% CI 0.61-1.77; *P*=.88; heterogeneity: *X*^2^_2_=3.91; *I*^2^=49%; *P*=.14).

#### Chronic Respiratory Diseases

Telerehabilitation had no significant effect on reducing respiratory-related hospitalizations (OR 1.03, 95% CI 0.79-1.35; *P*=.81), with no heterogeneity observed (*X*^2^_4_=3.8; *I*^2^=0%; *P*=.43).

#### Diabetes (Type II)

One included study reported a greater reduction in unplanned health care services usage among patients with diabetes in the telerehabilitation group compared to those in the control group [39]. However, another study found no significant differences [38].

#### Cancer

One study reported no significant difference in health-resource use between patients with cancer who received telerehabilitation and those in the control group [46].

### Risk of Bias

**Figure 3:**
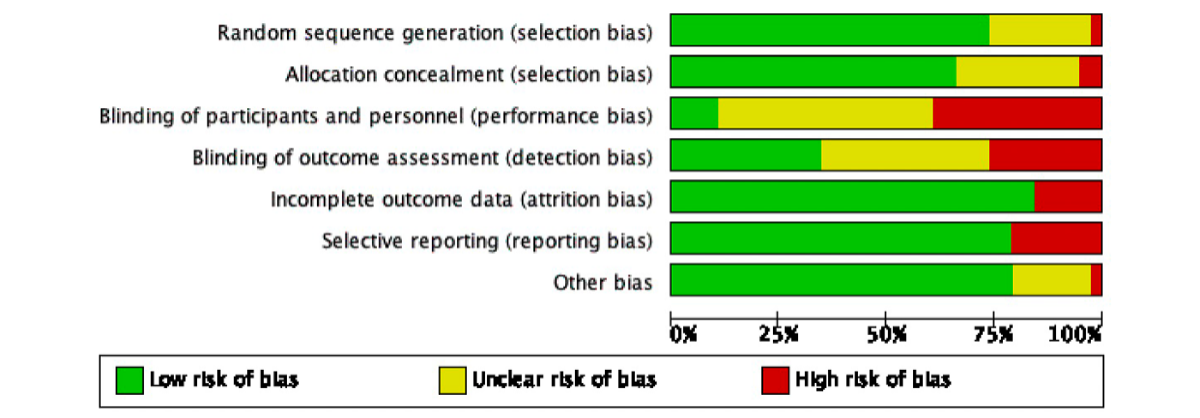
Risk of bias.

**Figure 4:**
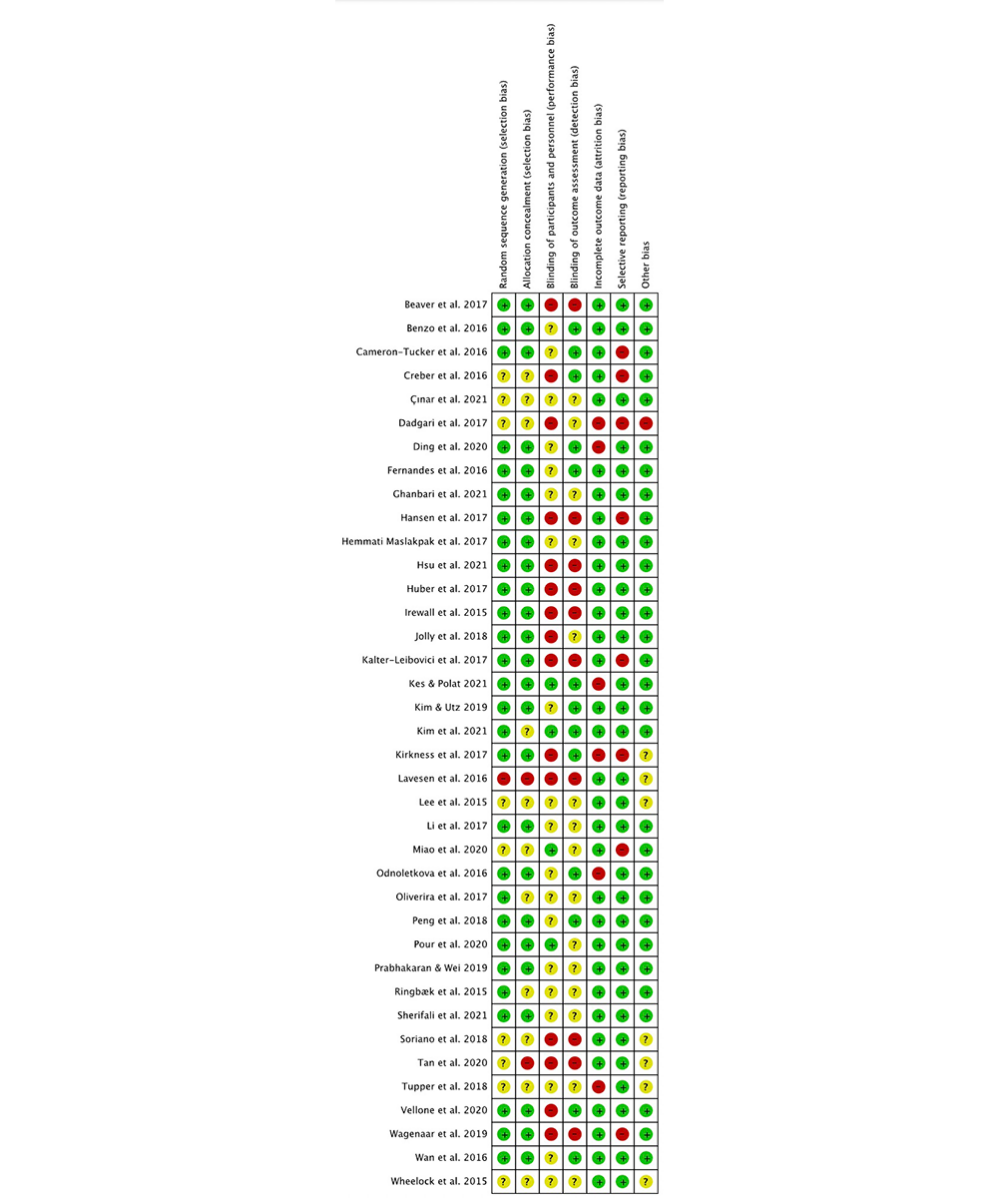
Risk of bias table.

A summary of the risk of bias in the studies is shown in Figure 3 and 4. The quality of the randomization and allocation concealment in most of the studies was good. However, due to the nature of telerehabilitation, blinding of participants and interventionists was difficult. There were 34 out of 42 studies rated as high or unclear risk regarding to blinding of participants and personnel (90%). In addition, 25 out of 42 studies were rated with high or unclear risk on blinding to outcome assessment (66%), while 12 studies had high risk on outcome reporting (29%).

## Discussion

### Principal Results

Given the limited resources in hospital settings and the ongoing COVID-19 pandemic, telerehabilitation seems to be a promising long-term approach to delivering continuous care from health care professionals to people with chronic diseases [49]. In this meta-analysis, it was found that patients with chronic disease experienced a significant improvement in their quality of life and self-care ability after receiving nurse-led telerehabilitation when compared with those who received a conventional in-person rehabilitation service. These improvements might have resulted from an increase in people’s knowledge of how to monitor their symptoms and in their ability to perform clinical assessments on their own after participating in a nurse-led telerehabilitation program [30,46]. Similar results were seen in previous reviews targeting community-dwelling patients [50] with heart failure [51], COPD [52], or cancer [53]. Thus, with these findings, telerehabilitation programs can be applied in community-based rehabilitation services, especially during the current COVID-19 pandemic. Although telerehabilitation is beneficial to the quality of life and self-care ability of patients with chronic disease, its effect on their psychological health and hospital admission is less certain. A previous review analyzing the effects of nurse-driven telerehabilitation programs found significant improvement in anxiety and depression level among patients with COPD [52]. In contrast, a few studies reported that telerehabilitation had no significant impact on the psychological health of those with chronic disease [18,23]. In addition, the effectiveness of telerehabilitation on reducing health resource use was also varied among previous reviews. A previous integrated review was not able to prove the effectiveness of telerehabilitation on reducing hospitalization rate among patients with heart failure [54]. Some reviews found significant reduction of nonplanned hospital admission [55] and emergency department visits [56]. In contrast, a few studies reported no significant differences on health-resource use for community-dwelling older adults [50], patients with diabetes [57], or those with heart failure [58]. These mixed findings might have resulted from differences in the characteristics of the patients and interventions in their respective nurse-led rehabilitation programs. Therefore, future reviews are needed to compare the effectiveness of telerehabilitation programs according to their different intervention characteristics, such as duration, delivery mode, and dosage.

Nurse telephone follow-ups were found to be the most common intervention component in nurse-led telerehabilitation programs, which was consistent with the finding in a previous study [59]. Telephone follow-ups were perceived to be by far the easiest way to ask health care providers questions about disease self-management, while not requiring any sophisticated devices [12,43,46]. By contrast, telemonitoring, another intervention component frequently used in telerehabilitation programs, was regarded as the least favorable by patients because of the frequent technical issues that arose during the transmission of data using wireless devices [18,60,61]. The patients were also concerned about the accuracy of the tools used in the in-home monitoring of vital signs and the wearable sensor [62]. In addition, the inability to use the monitoring tools and interpret their own health data were also common reasons for noncompliance in self-monitoring [18]. Telemonitoring is thus better implemented with adequate preintervention nursing education or training sessions for patients to familiarize themselves with the technological devices. Future research should also improve the quality of the telemonitoring system, including stability, accuracy, and security to increase patients’ confidence towards telemonitoring.

Despite their benefits, rehabilitation programs should not be provided solely via a telecare delivery mode. The lack of physical interaction can lead to difficulty in building a trusting nurse-patient relationship and hence lower the satisfaction of patients [26,42]. In addition, telerehabilitation may indeed increase the anxiety and depression levels of patients due to inexperience in using technology [23,63]. Patients with chronic disease may need regular face-to-face nurse consultations to solve the problems that they encounter during telerehabilitation. Supplementing telerehabilitation with face-to-face consultations allows for more comprehensive nursing assessments and physical examinations to be conducted [64].

Guided by the chronic care model, this review found that all included studies provided regular two-way interactions between patients and proactive health care providers. However, when abnormal findings or acute problems were identified, some studies did not provide evidence-based protocols for nurses to follow, which might have led to inaccuracy in clinical judgement and an increase in unnecessary hospital admission [65-67]. Therefore, a reliable guideline should be given to health care providers before the implementation of telerehabilitation programs.

Given the current pace of technological development, more advanced decision support systems can be improved with the aid of artificial intelligence (AI) [68]. Different decision support systems were developed in recent research for chronic disease management, most commonly for diagnosis, follow-up management, and treatment [69]. A previous study created an AI-based decision support system for enhancing shared decision-making in a pharmacotherapy regimen for patients with diabetes [70]. Based on patients’ clinical data, this AI-driven system can generate medication regimens with comprehensive information, including predicted success rate, risks and benefits, and medication costs. Another study adopted a machine-learning decision support system in telemonitoring to predict the risk of acute asthma exacerbation according to patients’ self-report symptoms, with timely alert notification to nurses when abnormalities were detected [71].

In addition to decision support systems, clinical information systems are another important component in the chronic care model that need to be considered in nurse-led telerehabilitation programs. The lack of a shared clinical information system among health care professionals has shown to increase the risk of medical errors [72]. With the use of technology, health records of patients can be shared electronically between patients and health care providers or among different health care disciplines. Evidence suggests that integrating electronic health records in community-based chronic disease care can effectively improve patients’ health outcomes and quality of health care services [73]. Although the use of electronic health records has been widely used and tested, it is limited to showing only objective physical indicators, such as blood glucose level and radiology reports. Recent studies have begun to allow patients to impute their subjective health complaints, such as symptoms and physical activity, into the electronic health records [74]. Nevertheless, concerns have been raised concerning data privacy issues when patients’ personal information were uploaded and stored on the internet [62,75]. Therefore, future research is needed to develop a cloud platform with a more advanced security system so as to prevent breaching of patient health data [76]. In addition, policy makers should regulate the storage and sharing of patient health information to third parties for medical follow-up and referral to ensure data privacy [77].

Chronic diseases are usually associated with functional impairment, which can reduce the ability of patients to adapt telerehabilitation. The evidence shows that patients with greater physical disabilities are less likely than their counterparts to comply with a telerehabilitation program [18,27,78]. To improve compliance, adequate preintervention training is needed on disease self-management and on the use of technological devices [79]. Among the 6 targeted chronic diseases, previous research suggested that cardiac diseases, chronic respiratory diseases, and stroke would cause higher functional disability in patients than would hypertension, diabetes, and cancer and thus compromise their ability to participate in telerehabilitation programs [80]. This may explain why improvement in the physical indicators examined in this review, including COPD exacerbation and physical disability level, was not significant among patients with cardiac diseases, chronic respiratory diseases, or stroke after participating in a nurse-led telerehabilitation program. In view of this, it is suggested that caregivers should be involved in assisting such patients to become engaged in telerehabilitation programs.

### Future Directions

Most older adults not only suffer from a single chronic disease, but also face the problem of multimorbidity. The prevalence of multimorbidity in China and the United States has been reported to be 49.4% and 59.6%, respectively [81,82]. Rehabilitation services for patients with multiple chronic diseases are more complex in nature than are those for patients with a single disease due to the interrelated pathophysiological pathways of chronic diseases [83]. The difficulty in interpreting symptoms and managing multiple medical regimens increases due to the overlapping signs and symptoms of these complex and interrelated chronic diseases [84]. It is thus crucial to conduct future studies to evaluate the effectiveness of nurse-led telerehabilitation programs among patients with multiple chronic conditions, as there are currently few such studies.

### Limitations

This review has several limitations. First, it only included papers written in English, so relevant studies reported in different languages were missed. Second, this review only included nurse-led telerehabilitation programs for the 6 most common chronic diseases. There was no coverage of telerehabilitation programs for those with other chronic diseases resulting in high functional disability, such as arthritis and neurological diseases. Third, the heterogeneity between studies was from moderate to high due to the differences in intervention characteristics, such as study duration, sample size, and technological devices used. Fourth, not all studies were included in the meta-analysis due to data incompleteness despite reviewers’ attempts to contact corresponding authors for relevant data.

### Conclusions

Although the meta-analysis showed that the programs led to a significant improvement in the quality of life and self-care ability of patients with various chronic diseases, it did not have an advantage over traditional face-to-face consultations with regard to anxiety, depression, or the number of hospital admissions. Guided by the chronic care model, the review showed that the usage of decision support and clinical information systems may facilitate the work of nurses in telerehabilitation programs. In addition, despite the commonality of multimorbidity, limited studies regarding the effectiveness of telerehabilitation programs targeting patients with multiple chronic diseases are available. Future research could focus on the use of telerehabilitation among these patients.

## Appendix

Multimedia Appendix 1 Search strategies.

Multimedia Appendix 2 Data extraction table.

Multimedia Appendix 3 Nurse follow-ups.

Multimedia Appendix 4 Telemonitoring.

Multimedia Appendix 5 Chronic care model.

Multimedia Appendix 6 PRISMA (Preferred Reporting Items for Systematic Reviews and Meta-Analyses) checklist.

## Abbreviations

AI: artificial intelligence
COPD: chronic obstructive pulmonary disease
MD: mean difference
OR: odds ratio
PICO: patient, intervention, comparison, and outcome
PRISMA: Preferred Reporting Items for Systematic Reviews and Meta-Analyses
PROSPERO: International Prospective Register of Systematic Reviews
SMD: standardized mean difference

## Data Availability

All data sets generated or analyzed during the current study are included in this published article and the supplementary materials.
