# Supplementary material for: Nurse-led Telehealth Intervention for Rehabilitation (Telerehabilitation) Among Community-Dwelling Patients With Chronic Diseases: Systematic Review and Meta-analysis
Source: J Med Internet Res. 2022 Nov 2;24(11):e40364. doi: 10.2196/40364 (PMC9669889; doi:10.2196/40364)
Supplement: Multimedia Appendix 1 [file jmir_v24i11e40364_app1.docx]

## Multimedia Appendix 1

Supplementary file 1. Searching Strategies

1. PubMed

| Searching Strategy | (("Telemedicine"[Mesh]) OR (technolog* OR telerehabilitation OR e-rehabilitation OR telecare OR telemedicine OR telehealth OR telecommunication OR telemonitor* OR telenursing OR tele-nursing OR ehealth OR e-health OR mhealth OR m-health OR telecoaching OR gerontechnology OR videoconferenc* OR teleconferenc* OR internet OR computer OR mobile OR phone OR smartphone OR telephone OR tablet OR email OR e-mail OR SMS OR apps OR applications OR “social media” OR wireless OR virtual OR remote OR distant))  AND  (("Chronic Disease"[Mesh]) OR ("Hypertension"[Mesh]) OR ("Myocardial Ischemia"[Mesh]) OR ("Heart Failure"[Mesh]) OR ("Asthma"[Mesh]) OR ("Pulmonary Disease, Chronic Obstructive"[Mesh]) OR ("Diabetes Mellitus"[Mesh]) OR ("Neoplasms"[Mesh]) OR ("Stroke"[Mesh]) OR (chronic OR “long term condition” OR hypertension OR hypertensive OR “high blood pressure” OR “elevated blood pressure” OR cardiovascular OR cardio-vascular OR CVD OR heart OR cardiac OR coronary OR cardiopulmonary OR respiratory OR pulmonary OR lung OR asthma OR “chronic obstructive pulmonary disease” OR COPD OR “chronic obstructive lung disease” OR diabetes OR diabetic OR “DM” OR hyperglycemia OR hyperglycaemia OR cancer OR carcinoma OR malignancy OR tumor OR tumour OR oncolog* OR stroke OR “cerebrovascular accident” OR CVA OR dementia OR alzheimers OR “cognitive impair*”))  AND  (("Nurses"[Mesh]) OR ("Nursing"[Mesh]) OR (nurs* OR nurse-led OR “nurse led” OR nurse-based OR “nurse based” OR interdisciplin* OR multi-disciplin* OR multidisciplin*)) |
| --- | --- |
| Filter Applied | - Results by Year: 2015-2021 - Article Type: Randomized Controlled Trial |

2. MEDLINE (via Ebscohost)

| Searching Strategy | (MH(“Telecommunication+”) OR MH(social media) OR (technolog* OR telerehabilitation OR e-rehabilitation OR telecare OR telemedicine OR telehealth OR telecommunication OR telemonitor* OR telenursing OR tele-nursing OR ehealth OR e-health OR mhealth OR m-health OR telecoaching OR gerontechnology OR videoconferenc* OR teleconferenc* OR internet OR computer OR mobile OR phone OR smartphone OR telephone OR tablet OR email OR e-mail OR SMS OR apps OR applications OR “social media” OR wireless OR virtual OR remote OR distant))  AND  ((MH "Chronic Disease+") OR (MH "Hypertension") OR (MH "Myocardial Ischemia+") OR (MH “Heart Failure+”) OR (MH "Asthma+") OR (MH "Pulmonary Disease, Chronic Obstructive+") OR (MH "Diabetes Mellitus+") OR (MH "Neoplasms+") OR (MH "Stroke+") OR (MH "Dementia+") OR (chronic OR “long term condition” OR hypertension OR hypertensive OR “high blood pressure” OR “elevated blood pressure” OR cardiovascular OR cardio-vascular OR CVD OR heart OR cardiac OR coronary OR cardiopulmonary OR respiratory OR pulmonary OR lung OR asthma OR “chronic obstructive pulmonary disease” OR COPD OR “chronic obstructive lung disease” OR diabetes OR diabetic OR “DM” OR hyperglycemia OR hyperglycaemia OR cancer OR carcinoma OR malignancy OR tumor OR tumour OR oncolog* OR stroke OR “cerebrovascular accident” OR CVA OR dementia OR alzheimers OR “cognitive impair*”))  AND  ((MH "Nurses+") OR (MH “Nursing+”) OR (nurs* OR nurse-led OR “nurse led” OR nurse-based OR “nurse based” OR interdisciplin* OR multi-disciplin* OR multidisciplin*)) |
| --- | --- |
| Filter Applied | - Scholarly (Peer Reviewed) Journals - Date of Publication: 2015-2021 - Publication Type: Randomized Controlled Trial |

3. CINAHL (via Ebscohost)

| Searching Strategy | ((MH "Telecommunications+") OR (technolog* OR telerehabilitation OR e-rehabilitation OR telecare OR telemedicine OR telehealth OR telecommunication OR telemonitor* OR telenursing OR tele-nursing OR ehealth OR e-health OR mhealth OR m-health OR telecoaching OR gerontechnology OR videoconferenc* OR teleconferenc* OR internet OR computer OR mobile OR phone OR smartphone OR telephone OR tablet OR email OR e-mail OR SMS OR apps OR applications OR “social media” OR wireless OR virtual OR remote OR distant))  AND  ((MH "Chronic Disease+") OR (MH "Hypertension") OR (MH "Myocardial Ischemia+") OR (MH”Heart Failure+”) OR (MH "Asthma+") OR (MH "Pulmonary Disease, Chronic Obstructive+") OR (MH "Diabetes Mellitus+") OR (MH "Glycemic Control") OR (MH "Neoplasms+") OR (MH "Rehabilitation, Cancer") OR (MH "Stroke+") OR (MH "Dementia+") OR (chronic OR “long term condition” OR hypertension OR hypertensive OR “high blood pressure” OR “elevated blood pressure” OR cardiovascular OR cardio-vascular OR CVD OR heart OR cardiac OR coronary OR cardiopulmonary OR respiratory OR pulmonary OR lung OR asthma OR “chronic obstructive pulmonary disease” OR COPD OR “chronic obstructive lung disease” OR diabetes OR diabetic OR “DM” OR hyperglycemia OR hyperglycaemia OR cancer OR carcinoma OR malignancy OR tumor OR tumour OR oncolog* OR stroke OR “cerebrovascular accident” OR CVA OR dementia OR alzheimers OR “cognitive impair*”))  AND  ((MH "Nurses+") OR (nurs* OR nurse-led OR “nurse led” OR nurse-based OR “nurse based” OR interdisciplin* OR multi-disciplin* OR multidisciplin*)) |
| --- | --- |
| Filter Applied | - Peer Reviewed - Date of Publication: 2015-2021 - Randomized Controlled Trial |

4. EMBASE

| Searching Strategy | ((technolog* OR telerehabilitation OR e-rehabilitation OR telecare OR telemedicine OR telehealth OR telecommunication OR telemonitor* OR telenursing OR tele-nursing OR ehealth OR e-health OR mhealth OR m-health OR telecoaching OR gerontechnology OR videoconferenc* OR teleconferenc* OR internet OR computer OR mobile OR phone OR smartphone OR telephone OR tablet OR email OR e-mail OR SMS OR apps OR applications OR “social media” OR wireless OR virtual OR remote OR distant))  AND  ((chronic OR “long term condition” OR hypertension OR hypertensive OR “high blood pressure” OR “elevated blood pressure” OR cardiovascular OR cardio-vascular OR CVD OR heart OR cardiac OR coronary OR cardiopulmonary OR respiratory OR pulmonary OR lung OR asthma OR “chronic obstructive pulmonary disease” OR COPD OR “chronic obstructive lung disease” OR diabetes OR diabetic OR “DM” OR hyperglycemia OR hyperglycaemia OR cancer OR carcinoma OR malignancy OR tumor OR tumour OR oncolog* OR stroke OR “cerebrovascular accident” OR CVA OR dementia OR alzheimers OR “cognitive impair*”))  AND  ((nurs* OR nurse-led OR “nurse led” OR nurse-based OR “nurse based” OR interdisciplin* OR multi-disciplin* OR multidisciplin*)) |
| --- | --- |
| Filter Applied | - Publication Year: 2015-2021 - Randomized Controlled Trial |

5. PsycINFO (via ProQuest)

| Searching Strategy | ((technolog* OR telerehabilitation OR e-rehabilitation OR telecare OR telemedicine OR telehealth OR telecommunication OR telemonitor* OR telenursing OR tele-nursing OR ehealth OR e-health OR mhealth OR m-health OR telecoaching OR gerontechnology OR videoconferenc* OR teleconferenc* OR internet OR computer OR mobile OR phone OR smartphone OR telephone OR tablet OR email OR e-mail OR SMS OR apps OR applications OR “social media” OR wireless OR virtual OR remote OR distant))  AND  ((chronic OR “long term condition” OR hypertension OR hypertensive OR “high blood pressure” OR “elevated blood pressure” OR cardiovascular OR cardio-vascular OR CVD OR heart OR cardiac OR coronary OR cardiopulmonary OR respiratory OR pulmonary OR lung OR asthma OR “chronic obstructive pulmonary disease” OR COPD OR “chronic obstructive lung disease” OR diabetes OR diabetic OR “DM” OR hyperglycemia OR hyperglycaemia OR cancer OR carcinoma OR malignancy OR tumor OR tumour OR oncolog* OR stroke OR “cerebrovascular accident” OR CVA OR dementia OR alzheimers OR “cognitive impair*”))  AND  ((nurs* OR nurse-led OR “nurse led” OR nurse-based OR “nurse based” OR interdisciplin* OR multi-disciplin* OR multidisciplin*))  AND  ((“randomi?ed control* trial” OR RCT OR “control* trial” OR “random* allocat*” OR “random* assign*” OR “randomi?ed control* clinical trial” OR “clinical control* trial” OR “randomi?ed control* study” OR randomi?ed)) |
| --- | --- |
| Filter Applied | - Date: 2015-2021 - Peer reviewed |

6. Cochrane Central Register of Controlled Trials (CENTRAL)

| Searching Strategy | #1: MeSH descriptor: [Telemedicine] explode all trees  #2: (technolog* OR telerehabilitation OR e-rehabilitation OR telecare OR telemedicine OR telehealth OR telecommunication OR telemonitor* OR telenursing OR tele-nursing OR ehealth OR e-health OR mhealth OR m-health OR telecoaching OR gerontechnology OR videoconferenc* OR teleconferenc* OR internet OR computer OR mobile OR phone OR smartphone OR telephone OR tablet OR email OR e-mail OR SMS OR apps OR applications OR “social media” OR wireless OR virtual OR remote OR distant)  #3: MeSH descriptor: [Chronic Disease] explode all trees  #4: MeSH descriptor: [Hypertension] this term only  #5: MeSH descriptor: [Heart Failure] explode all trees  #6: MeSH descriptor: [Myocardial Ischemia] explode all trees  #7: MeSH descriptor: [Asthma] explode all trees  #8: MeSH descriptor: [Pulmonary Disease, Chronic Obstructive] explode all trees  #9: MeSH descriptor: [Diabetes Mellitus] explode all trees  #10: MeSH descriptor: [Neoplasms] explode all trees  #11: MeSH descriptor: [Stroke] explode all trees  #12: MeSH descriptor: [Dementia] explode all trees  #13: (chronic OR “long term condition” OR hypertension OR hypertensive OR “high blood pressure” OR “elevated blood pressure” OR cardiovascular OR cardio-vascular OR CVD OR heart OR cardiac OR coronary OR cardiopulmonary OR respiratory OR pulmonary OR lung OR asthma OR “chronic obstructive pulmonary disease” OR COPD OR “chronic obstructive lung disease” OR diabetes OR diabetic OR “DM” OR hyperglycemia OR hyperglycaemia OR cancer OR carcinoma OR malignancy OR tumor OR tumour OR oncolog* OR stroke OR “cerebrovascular accident” OR CVA OR dementia OR alzheimers OR “cognitive impair*”)  #14: MeSH descriptor: [Nurses] explode all trees  #15: MeSH descriptor: [Nursing] explode all trees  #16: (nurs* OR nurse-led OR “nurse led” OR nurse-based OR “nurse based” OR interdisciplin* OR multi-disciplin* OR multidisciplin*)  #17: #1 OR #2  #18: #3 OR #4 OR #5 OR #6 OR #7 OR #8 OR #9 OR #10 OR #11 OR #12 OR #13  #19: #14 OR #15 OR #16  #20: #17 AND #18 AND #19 |
| --- | --- |
| Filter Applied | - CENTRAL Trials only - Original publication year: 2015-2021 - Trial |
