# Supplementary material for: Nurse-led Telehealth Intervention for Rehabilitation (Telerehabilitation) Among Community-Dwelling Patients With Chronic Diseases: Systematic Review and Meta-analysis
Source: J Med Internet Res. 2022 Nov 2;24(11):e40364. doi: 10.2196/40364 (PMC9669889; doi:10.2196/40364)
Supplement: Multimedia Appendix 2 [file jmir_v24i11e40364_app2.docx]

## Multimedia Appendix 2

Supplementary file 2. Data extraction table

1. Hypertension

| **Author**  **(Year of publication)** | **Study Location** | **Study Population** | **Provider** | **Duration** | **Intervention Group (IG)** | | **Control Group (CG)** | **Data Collection Timepoint** | **Outcome Variables** | **Outcome Measures** | **Result** |
| --- | --- | --- | --- | --- | --- | --- | --- | --- | --- | --- | --- |
|  |  |  |  |  | **Technological**  **(a) Delivery mode**  **(b) Content**  **(c) Frequency/Duration** | **Non-technological**  **(a) Delivery mode**  **(b) Content**  **(c) Frequency/Duration** |  |  |  |  |  |
| Dadgari et al. (2017) | Iran | Patients (aged 18-65) diagnosed with hypertension and using antihypertensive agents  (n=65)  (Mean age: 58.48)  **IG:** n=32,  Mean Age: NA  **CG:** n=33,  Mean Age: NA | Nurses | 8 weeks | **Telenursing:**  **(1) Nurse telephone follow-ups**  (a) Telephone  (b) Address questions on blood pressure self-monitoring    (c) Twice during the first 2 weeks and once during the next 2 weeks | **(1) Education session**  (a) Face-to-face  (b) Set behavioral goals, action plan + Educate on blood pressure self-management (involving patient's close relative)  (c) Once, 45 mins  **(2) Written pamphlet**  (b) NA | (1) Same education session as IG  (2) Written pamphlet | T0: baseline  T1: 8 weeks  T2: 12 weeks (follow-up) | (1) Quality of life | (1) SF-36 Questionnaire | 1. QoL of IG was significantly higher than CG   [T1: (IG) 176.96±8.25 vs (CG) 173.47±8.08; p=0.01]  [T2: (IG) 179.06±8.63 vs (CG) 172.03±9.21; p=0.0001] |
| Kes & Polat (2021) | Turkey | Patients (aged 40-64) diagnosed with primary hypertension  (n=92)  (Mean Age: NA)  **IG:** n=46 (Analyzed: 39), Mean Age: 54.9  **CG:** n=46 (Analyzed: 38), Mean Age: 52.2 | Nurses | 12 weeks | **(1) Nurse telephone monitoring**  (a) Telephone  (b) Blood pressure monitoring + Education on medication adherence  (c) Weeks 2, 4, 6, 8, 12 (Total: 5 calls)  **(2) Text-messaging**  (a) SMS  (b) Personalized medication reminders + informational SMS  (c) Weeks 1, 3, 5, 7, 9 (Total: 3 SMS weekly) | **(1) Training session**  (a) NA  (b) Hypertension self-management  (c) Once  **(2) Educational brochure and BP monitoring card**  (b) Blood pressure monitoring, medication adherence | (1) Same training session as IG  (2) Educational brochure and BP monitoring card  (3) Usual care in health center | T0: baseline  T1: 12 weeks | (1) Blood pressure | (1) Systolic and diastolic blood pressure | 1. Mean BP of IG was significantly lower than CG   SBP  [T1: (IG) 136.08±6.91 vs (CG) 149.24±8.42; p=0.001]  **Included in meta-analysis:**  (MD=13.16; 95% CI: 9.71 to 16.61)  DBP  [T1: (IG) 86.56±4.84 vs (CG) 93.61±5.57; p=0.001]  **Included in meta-analysis:**  (MD=7.05; 95% CI: 4.72 to 9.38) |
| Miao et al. (2020) | China | Patients (aged above 18) diagnosed with hypertension  (n=156)  (Mean Age: 67.4)  **IG:** n=78,  Mean Age: 68.9  **CG:** n=78,  Mean Age: 66.8 | Nurses | 12 weeks | **(1) Nurse telephone follow-ups**  (a) Telephone  (b) Blood pressure monitoring + Referral if needed  (c) Biweekly, Average 10 mins | **(1) Pre-intervention training program (For nurses)**  (b) Hypertension management  (c) 36 hours  **(2) Nurse home visit**  (a) Face-to-face  (b) Initial nursing assessment + Education + Case management  (c) Once, 60 mins | (1) Free annual health check  (2) Health education leaflets  (3) Usual follow-up with general practitioners | T0: baseline  T1: 12 weeks  T3: 16 weeks (follow-up) | (1) Blood pressure  (2) Self-care behaviors | (1) Systolic and diastolic blood pressure  (2)  (i) Adherence to anti-hypertensive drugs  (ii) Non-pharmacological behaviors | 1. IG had significantly decreased blood pressure in T1   SBP  [T1-T0: (IG) 15.03±23.75; p=0.032]  DBP  [T1-T0: (IG) 8.54±8.86; p=0.026]  (2)  (i) No significant difference  (ii) Non-pharmacological behavior adherence of IG was significantly higher than CG [(IG vs CG) T1: p=0.000, T2: p=0.049] |
| Pour et al. (2020) | Iran | Patients (aged 35-64) diagnosed with hypertension  (n=63)  (Mean Age: NA)  **IG:**  ISMS: n=21, Mean Age: 54.71    NISMS: n=21, Mean Age: 55.95  **CG:** n=21, Mean Age: 56.71 | Nurses | 4 months | **ISMS (Interactive SMS):**  **(1) Non-interactive SMS**  (a) SMS  (b) Hypertension management and lifestyle modifications  (c) 4 messages a week (Total: 64)  **(2) Interactive SMS**  (a) SMS  (b) Communicate with nurse to answer their questions  (c) At least twice a month  **NISMS (Non-interactive SMS):**  Same intervention as ISMS but without communication with nurses | **All IG**  **(1) Education session**  (a) NA  (b) Hypertension self-management and healthy lifestyle  (c) Once  **(2) Educational booklet**  (b) Blood pressure monitoring and healthy lifestyle | Receive all SMS messages post-test | T0: baseline  T1: 1 month  T2: 2 months  T3: 3 months  T4: 4 months | (1) Blood pressure | (1) Systolic and diastolic blood pressure | 1. Blood pressure in both IG had decreased (ISMS: p<0.001; NISMS: p=0.02), while blood pressure in CG had no statistically significant change (p=0.27)   SBP  [T4: (ISMS) 133.14±14.52 vs (NISMS) 139.52±9.79 vs (CG) 137.71±20.9; p=0.42]  **Included in meta-analysis (ISMS vs CG):**  (MD=4.57; 95% CI: -6.31 to 15.45)  DBP  [T4: (ISMS) 9.148±5.72 vs (NISMS) 89±5.97 vs (CG) 86.67±13.97; p=0.26]  **Included in meta-analysis (ISMS vs CG):**  (MD=-4.81; 95% CI: -11.27 to 1.65) |

2. Cardiac Diseases

| **Author**  **(Year of publication)** | **Study Location** | **Study Population** | **Provider** | **Duration** | **Intervention Group (IG)** | | **Control Group (CG)** | **Data Collection Timepoint** | **Outcome Variables** | **Outcome Measures** | **Result** |
| --- | --- | --- | --- | --- | --- | --- | --- | --- | --- | --- | --- |
|  |  |  |  |  | **Technological**  **(a) Delivery mode**  **(b) Content**  **(c) Frequency/Duration** | **Non-technological**  **(a) Delivery mode**  **(b) Content**  **(c) Frequency/Duration** |  |  |  |  |  |
| Creber et al. (2016) | US | Patient (aged 18 or above) diagnosed with symptomatic heart failure (NYHA II-IV)  (n=67)  (Mean Age: 62)  **IG:** n=41,  Mean Age: 60  **CG:** n=26,  Mean Age: 63 | Nurses | 90 days | **Motivational Interviewing Tailored Intervention for Heart Failure (MITI-HF):**  **(1) Nurse telephone follow-ups**  (a) Telephone  (b) Reinforcement after motivational interviewing  (c) Total: 3-4 over 90 days | **(1) Nurse home visit**  (a) Face-to-face  (b) Initial motivational interviewing on disease self-care + SCHFI score for decision support on patient’s goal  (c) Once | (1) Usual care  (2) Educational materials on HF self-care | T0: baseline  T1: 90 days | (1) Self-care maintenance  (2) Physical HF symptoms  (3) Quality of life | (1) Self-Care of Heart Failure Index (SCHFI) v.6.2  (2) Heart Failure Somatic Perception Scale (HFSPS)  (3) Kansas City Cardiomyopathy Questionnaire (KCCQ) | 1. No statistically significant change   Self-care maintenance  [T0-T1: (IG) 19.7±16.0 vs (CG) 12.1±18.3; p=0.08]  Self-care confidence  [T0-T1: (IG) 26.6±20.8 vs (CG) 21.6±16.8; p=0.31]   1. No statistically significant change   [T0-T1: (IG) -2.8±16.8 vs (CG) -0.73±17.1; p=0.63]   1. No statistically significant change   [T0-T1: (IG) 10.8±28.2 vs (CG) 4.81±21.4; p=0.36] |
| Ding et al. (2020) | Australia | Patients (aged 18 or above) diagnosed with chronic heart failure (HF)  (n=184)  (Mean Age: 70.1)  **IG:** n=91,  Mean Age: 69.5  **CG:** n=93,  Mean Age: 70.8 | Nurses | 6 months | **Innovative Telemonitoring Enhanced Care Program for CHF (ITEC-CHF):**  **(1) Telemonitoring**  (a) Electronic weight scale + Tablet using Bluetooth function  (b) Remote body weight monitoring: auto transmission of data from weight scale to tablet 🡪 Decision support system: generate alert to nurses  (c) Measured by patient daily  **(2) Nurse telephone support**  (a) Telephone  (b) Provide support according to alerts produced from a decision support system  (c) Weekdays | **(1) Paper-based diary**  (b) NA  **(2) Educational booklet**  (b) "Living Well with Chronic Heart Failure" | (1) Paper-based diary  (2) Same educational booklet as IG  (3) Weight self-monitoring using an electronic weight scale daily. Data were collected by a nurse in a home visit | T0: baseline  T1: 6 months | (1) Health-related quality of life  (2) Psychological state  (3) CHF-related hospitalization | (1) EuroQol-5D (EQ-5D)  (2) Cardiac depression scale short form 2  (3) CHF-related hospitalization | 1. No statistically significant difference   [T0-T1: (IG) 4.05±15.95 vs (CG) 1.10±14.24; p=0.13]  **Included in meta-analysis:**  (SMD=0.30; 95% CI: 0.01 to 0.59)   1. No statistically significant difference   **Included in meta-analysis:**  (SMD=0.67; 95% CI: 0.23 to 1.12)   1. No statistically significant difference   [T1: (IG) 15 vs (CG) 8; p=0.24]  **Included in meta-analysis:**  (OR=2.10; 95% CI: 0.84 to 5.22) |
| Hsu et al. (2021) | Taiwan | Patients (aged 20 or above) diagnosed with heart failure (NYHA I-III)  (n=82)  (Mean Age: 68.54)  **IG:** n=41,  Mean Age: 69.32  **CG:** n=41,  Mean Age: 67.76 | Senior Nurses | 4 weeks | **(1) Nurse telephone follow-ups**  (a) Telephone  (b) Review self-care behavior and personal goals  (c) Twice per week (Total: 8 calls), 15-20 mins | **(1) Education session**  (a) Face-to-face  (b) Heart failure self-care  (c) Once, 20-30 mins  **(2) Self-regulation handbook**  (b) Heart failure self-care | Usual routine care | T0: baseline  T1: 4 weeks  T2: 8 weeks (follow-up) | (1) Self-care behavior | (1) Self-Care of Heart Failure Index (SCHFI) v.6.2 | 1. IG had significantly greater improvement than CG   Self-care maintenance  [T1: (IG) 51.25±16.91 vs (CG) 50.68±20.43]  [T2: (IG) 52.31±16.77 vs (CG) 52.79±24.97]  [Group*Time: (T1) B=3.74; p=0.01; (T2) B=2.68; p=0.312]  **Included in meta-analysis:**  (MD=-0.48; 95% CI: -9.69 to 8.73)  Self-care management  [T1: (IG) 41.00±18.17 vs (CG) 28.33±11.63]  [T2: (IG) 31.30±14.40 vs (CG) 28.33±11.63]  [Group*Time: (T1) B=6.33; p=0.004; (T2) B=6.97; p=0.030]  **Included in meta-analysis:**  (MD=2.97; 95% CI: -2.70 to 8.64)  Self-care confidence  [T1: (IG) 59.53±26.85 vs (CG) 51.26±25.62]  [T2: (IG) 60.48±26.50 vs (CG) 51.12±25.70]  [Group*Time: (T1) B=5.15; p=0.003; (T2) B=6.24; p=0.001]  **Included in meta-analysis:**  (MD=9.36; 95% CI: -1.94 to 20.66) |
| Huber et al. (2017)  Henriksson et al. (2021) | Sweden | Patients experienced acute coronary syndrome (ACS)  (n=962)  (Mean Age: NA)  **IG:** n=486 (Analyzed: 406), Mean Age: 67.3  **CG:** n=476 (Analyzed: 391), Mean Age: 68.4 | Nurses + Physician | 36 months | **NAILED-ACS Trial:**  **(1) Nurse telephone session**  (a) Telephone  (b) Education on medication adherence and healthy lifestyle + Motivational interviewing + Contact physician for medication titration if target-level not reached (BP<140/90mmHg, LDL-C<1.8)  (c) NA (First call at 1 month after discharge and after each follow-up 🡪 Additional nurse telephone follow-up 1 month after medication titration) | (1) Follow-up on blood pressure and blood lipids every 12 months | Usual follow-up in cardiology clinic | T0: baseline  T1: 1 month  T2: 12 months  T3: 24 months  T4: 36 months | (1) Mean blood pressure  (2) Mean LDL-C | (1) Mean systolic and diastolic blood pressure  (2) Mean LDL-C | 1. IG had significantly lower SBP and DBP than CG   SBP  [T1: (IG) 124.9±13.3 vs (CG) 131.8±18.9; p<0.001]  [T2: (IG) 131.5±17.1 vs (CG) 133.0±19.2; p=0.24]  [T4: (IG) 129.4 vs (CG) 133.5; p<0.001]  DBP  [T1: (IG) 73.7±9.5 vs (CG) 77.7±11.2; p<0.001]  [T2: (IG) 76.0±10.2 vs (CG) 78.2±11.3; p=0.007]  [T4: (IG) 75.7 vs (CG) 78.6; p<0.001]   1. IG had significantly lower mean LDL-C than CG   [T1: (IG) 1.9±0.46 vs (CG) 2.3±0.87; p<0.001]  [T2: (IG) 2.1±0.74 vs (CG) 2.4±0.96; p<0.001]  [T4: (IG) 2.14 vs (CG) 2.42; p<0.001] |
| Kalter-Leibovici et al. (2017) | Israel | Patients (aged 18 or above) diagnosed with chronic heart failure (NYHA II-IV)  (n=1360)  (Mean Age: 70.7)  **IG:** n=682,  Mean Age: 70.8  **CG:** n=678,  Mean Age: 70.7 | Nurses | Mean FU:  2.7 years  (0-5 years) | **(1) Remote nurse follow-ups**  (a) Telephone/ video sessions  (b) Self-management support for patients and caregivers + Signs and symptom monitoring + Medication titration  (c) Once a week initially, further modified according to patients  **(2) Telemonitoring**  (a) Home telemonitoring device (Medic4All®)  (b) Telemonitoring data (body weight, BP, P) sent to electronic medical record + Automatic alerts generated to nurses if there were abnormal data  (c) Measured by patient daily | **(1) Follow-up visits**  (a) Face-to-face  (b) Health failure center: cardiologists, nurses, dietitians, social workers counseling  (c) At least once every 6 months | Usual follow-up at heart failure centers every 6 months | T0: baseline  T1: every 6 months until death or end of study | (1) HF-related hospital admission  (2) NYHA classification  (3) Health-related quality of life  (4) Depression | (1) HF-related hospital admission  (2) NYHA classification  (3) 36-item short-form questionnaire (SF-36)  (4) 9-item patient health depression scale (PHQ-9) | 1. No significant difference   Percentage of patients admitted  [(IG) 56.9% vs (CG) 57%]  Average number of HF-related hospital admissions   - No significant difference (p=0.051)   In-hospital days   - No significant difference   **Included in meta-analysis:**  (OR=0.99; 95% CI: 0.80 to 1.23)   1. IG were more likely to improve than CG (Adjusted OR=2.477; 95% CI: 1.107-1.972) 2. IG were more likely to improve than CG   Physical: (Adjusted OR=1.531; 95% CI: 1.165-2.011)  Mental: (Adjusted OR=1.571; 95% CI: 1.253-1.971)   1. IG were less likely to experience moderate-to-severe depression symptoms than CG (adjusted OR=0.688; 95% CI: 0.528-0.897) |
| Oliveira et al. (2017) | Brazil | Patients (aged 18 or above) diagnosed with heart failure (NYHA I-III)  (n=36)  (Mean Age: NA)  **IG:** n=19,  Mean Age: 60.5  **CG:** n=17,  Mean Age: 60 | Nurses | 4 months | **(1) Nurse telephone follow-ups**  (a) Telephone  (b) Answer queries and education on HF and its management (guided by “Guide for telephone-based monitoring”)  (c) Total: 12 calls | **(1) Education session**  (a) Face-to-face  (b) HF self-management  (c) Once  **(2) Explanatory booklet**  (b) HF self-care | (1) Same education sessions as IG  (2) Explanatory booklet on HF self-care | T0: baseline  T1: 2 months  T2: 4 months | (1) Self-care | (1) European Heart Failure Behavior Scale (EHFScBS) Questionnaire  🡪 lower score, better self-care | 1. IG had more significantly improved self-care than CG   [T1: (IG) 26.3±6.8 vs (CG) 30.2±5.7]  [T2: (IG) 25.4±6.6 vs (CG) 29.5±4.8; p=0.04] |
| Peng et al. (2018) | China | Patients (aged 18 or above) diagnosed with chronic heart failure (CHF) (NYHA I-III)  (n=98)  (Mean Age: 66.3)  **IG:** n=49 (Analyzed: 42), Mean Age: NA  **CG:** n=49 (Analyzed: 41), Mean Age: NA | Multidisciplinary team (PT, nurses) | 8 weeks | **Telehealth Training Exercise Program:**  **(1) Exercise training**  (a) Online videoconferencing  (b) Exercise training (adjust exercise intensity according to heart rate)  (c) Stage 1s(wks 1-4): 3 per week (Total: 12), 20 mins each  Stage 2 (wks 5-8): 5 per week (Total: 20), 30 mins each  **(2) PT follow-ups**  (a) QQ and WeChat  (b) PT supervision + Monitoring of training intensity  (c) Throughout stages 1 and 2  **(3) Nurse follow-ups**  (a) Telephone or QQ and WeChat  (b) Solve patients’ enquiries + Referral if needed  (c) Weekly | **(1) Exercise training lecture**  (a) Face-to-face  (b) Skill demonstration  (c) Once  **(2) Educational brochure**  (b) HF self-care + Physical training information | **Usual care:**  (1) Discharge education  (2) Regular follow-up | T0: baseline  T1: 8 weeks  T2: 24 weeks (follow-up) | (1) Health-related quality of life  (2) NYHA classification  (3) Anxiety and depression | (1) Minnesota Living with Heart Failure Questionnaire (MLHFQ)  -> lower score, better QoL  (2) NYHA classification  (3) Hospital Anxiety and Depression Scale (HADS)  -> lower score, less anxiety and depression | 1. IG was more significantly improved than CG   [T1: (IG) 43.11±8.76 vs (CG) 49.20±12.44]  [T2: (IG) 42.32±8.83 vs (CG) 49.63±12.39]  [Between group: F=8.272, p=0.005]  [Changes over time: F=50.05, p=0.000]  [Group-time interaction: F=79.73, p=0.000]  **Included in meta-analysis:**  (SMD=0.67; 95% CI: 0.23 to 1.12)   1. No significant difference   [T1: (IG) 2.28±0.889 vs (CG) 2.18±0.697]  [T2: (IG) 2.29±0.890 vs (CG) 2.18±0.697]  [Between group: F=0.418, p=0.519]  [Changes over time: F=3.038, p=0.084]  [Group-time interaction: F=0.338, p=0.562]   1. No significant difference   Anxiety  [T1: (IG) 6.56±0.965 vs (CG) 6.77±0.743]  [T2: (IG) 6.53±0.927 vs (CG) 6.82±0.727]  [Between group: F=3.236, p=0.075]  [Changes over time: F=2.549, p=0.87]  [Group-time interaction: F=1.829, p=0.116]  Depression  [T1: (IG) 6.64±0.973 vs (CG) 6.70±0.924]  [T2: (IG) 6.58±0.979 vs (CG) 6.76±0.856]  [Between group: F=3.397, p=0.117]  [Changes over time: F=2.682, p=0.97]  [Group-time interaction: F=2.708, p=0.050]  **Included in meta-analysis:**  (SMD=0.19; 95% CI: -0.24 to 0.63) |
| Vellone et al. (2020) | Italy | Patients diagnosed with heart failure (HF) (NYHA II-IV) and their caregivers  (Dyad: n=510)  (Median age: patient: 74; caregiver: 55)  **IG:**  Arm 1:  n=155 (dyad), **Median** Age: Patient: 74; Caregiver: 54  Arm 2:  n=177 (dyad), **Median** Age: Patient: 73; Caregiver: 57  **CG:** n=178 (dyad), **Median** Age: Patient: 75; Caregiver: 53 | Nurses | 2 months | **Motivational interviewing to improve self-care in heart failure patients (MOTIVATE-HF):**  **Arm 1: MI only for patients**  **(1) Nurse telephone intervention**  (a) Telephone  (b) MI intervention for patients  (c) Total: 3, 15 mins  **Arm 2: MI for patients and caregivers**  **(1) Nurse telephone intervention**  (a) Telephone  (b) MI intervention for patients and caregivers separately  (c) Total: 3, 15 mins | **Both Arms 1 and 2:**  **(1) MI session**  (a) Face-to-face  (b) MI intervention  (c) Once, 60 mins  **(2) Educational material**  (b) HF self-care | (1) Education material for HF self-care  (2) Usual care (medical checkup every 6-12 months) | T0: baseline  T1: 3 months (follow-up)  T2: 6 months (follow-up)  T3: 9 months (follow-up)  T4: 12 months (follow-up) | (1) Self-care | (1) Self-Care of Heart Failure Index (SCHFI) v.6.2 | 1. Both IG had more significantly improved self-care maintenance than CG   Self-care maintenance  [T4-T0: (Arm 1) 21.19±16.71 vs (Arm 2) 18.84±20.74 vs (CG) 14.65±18.89; p=0.0480]  Score>=70: [T4: (Arm 1) 32.9% vs (Arm 2) 34.8% vs (CG) 18.4%; p=0.0138]  **Included in meta-analysis:**  (MD=6.23; 95% CI: 2.37 to 10.09)  Self-care management  [T4-T0: (Arm 1) 18.17±19.59 vs (Arm 2) 26.72±23.16 vs (CG) 15±18.76; p=0.1009] 🡪 No significant difference  Score>=70: [T4: (Arm 1) 29.7% vs (Arm 2) 30.8% vs (CG) 16.2%; p=0.1086] 🡪 No significant difference  **Included in meta-analysis:**  (MD=9.19; 95% CI: 4.87 to 13.51)  Self-care confidence  [T4-T0: (Arm 1) 17.67±20.73 vs (Arm 2) 15.48±27.28 vs (CG) 12.51±27.56; p=0.2865] 🡪 No significant difference  Score>=70: [T4: (Arm 1) 42.5% vs (Arm 2) 39.3% vs (CG) 30.3%; p=0.1194] 🡪 No significant difference  **Included in meta-analysis:**  (MD=3.50; 95% CI: -0.99 to 7.99) |
| Wagenaar et al. (2019) | Netherlands | Patients (aged 18 or above) with heart failure (HF)  (n=450)  (Mean Age: 66.8)  **IG:**  Arm 1: n=150, Mean Age: 66.6  Arm 2: n=150,  Mean Age: 66.7  **CG:** n=150,  Mean Age: 66.9 | HF nurses | 12 months | **Arm 1: E-health adjusted care pathway group**  **(1) Telemonitoring**  (a) e-vita platform  (b) Telemonitoring on body weight, BP, and HR + Pre-specified limits were set through shared decision making with HF nurses and patients + Alerts sent to nurses if abnormalities noted  (c) Specific timepoints everyday  **(2) Nurse telephone follow-ups**  (a) Telephone  (b) Manage signs and symptoms  (c) When any abnormalities noted  **(3) Reminders**  (a) Emails  (b) Reminders to use the intervention  (c) Monthly  **Arm 2: Website Group**  **(1) 'Heartfailurematters.org'**  (a) Website  (b) Information to support self-care by patients and their caregivers  (c) NA  **(2) Reminders**  (a) Emails  (b) Reminders to use the website  (c) Every 3 months | **Arm 2: Website Group**  **(1) Information leaflet**  (b) NA | Routine consultation from a cardiologist and an HF nurse (average 4 per year) | T0: baseline  T1: 3 months  T2: 6 months  T3: 12 months | (1) Self-care  (2) Health-related Quality of Life (HRQoL)  (3) HF-related hospitalizations | (1) European Heart Failure Self-care Behaviour (EHFScB) Scale  (2) Minnesota Living with HF Questionnaire  -> lower score, better QoL  (3) HF-related hospitalizations | 1. IG had significantly higher self-care than CG in T1, but no significant difference at T2 and T3   [T1: (Arm 1) 78.2 vs (Arm 2) 73.5 vs (CG) 70.8; p<0.001]  [T2: (Arm 1) 78.6 vs (Arm 2) 74.7 vs (CG) 74.2; p=0.070]  [T3: (Arm 1) 76.1 vs (Arm 2) 72.1 vs (CG) 72.7; p=0.184]   1. IG had significantly higher HRQoL than CG in T1 and T2, but no significant difference at T3   [T1: (Arm 1) 19.0 vs (Arm 2) 26.5 vs (CG) 22.8; p=0.029]  [T2: (Arm 1) 21.0 vs (Arm 2) 26.0 vs (CG) 20.4; p=0.070]  [T3: (Arm 1) 25.5 vs (Arm 2) 28.3 vs (CG) 26.5]   1. No significant difference   [(Arm 1) n=7 vs (CG) n=12; HR=0.57; 95% CI: 0.23-1.45]  [(Arm 2) n=8 vs (CG) n=12; HR: 0.65; 95% CI: 0.27-1.60]  **Included in meta-analysis:**  (OR=0.56; 95% CI: 0.22 to 1.47) |

3. Chronic Respiratory Diseases

| **Author**  **(Year of publication)** | **Study Location** | **Study Population** | **Provider** | **Duration** | **Intervention Group (IG)** | | **Control Group (CG)** | **Data Collection Timepoint** | **Outcome Variables** | **Outcome Measures** | **Result** |  |  |
| --- | --- | --- | --- | --- | --- | --- | --- | --- | --- | --- | --- | --- | --- |
|  |  |  |  |  | **Technological**  **(a) Delivery mode**  **(b) Content**  **(c) Frequency/Duration** | **Non-technological**  **(a) Delivery mode**  **(b) Content**  **(c) Frequency/Duration** |  |  |  |  |  |  |  |
| Benzo et al. (2016)  Benzo & McEvoy (2019) | US | Patients (aged 40 or above) with history of COPD exacerbation (current or ex-smoker)  (n=215)  (Mean Age: NA)  **IG:** n=108,  Mean Age: 67.9  **CG:** n=107, Mean Age: 68.1 | Nurses and respirator therapists | NA | **(1) Follow-up telephone calls**  (a) Telephone  (b) Motivational interviewing on lifestyle changes  (c) Weekly for the first 3 months, then monthly after 3 months | **(1) Education session**  (a) Face-to-face  (b) Provide a written emergency plan for COPD exacerbation + Education on COPD self-management  (c) Once, 2 hours  **(2) Written booklet**  (b) "Living a Healthy Life with Chronic Conditions" | (1) Usual care  (2) Written booklet: "Living a Healthy Life with Chronic Conditions" | T0: baseline  T1: 1 month  T2: 3 months  T3: 6 months  T4: 9 months  T5: 12 months | (1) Rate of COPD-related hospitalization  (2) Disease specific quality of life | (1) Rate of COPD-related hospitalization  (2) Chronic Respiratory Disease Questionnaire (CRQ) | 1. Rate of COPD-related hospitalizations in IG was significantly lower than CG at T1, T2, and T3, but no significant difference at T4 and T5   [T1: (IG) 1.9% vs (CG) 9.4%; ARR=7.5%; p=0.0174]  [T2: (IG) 9.4% vs (CG) 20.4%; ARR=11.0%; p=0.0280]  [T3: (IG) 15.4% vs (CG) 27.7%; ARR=11.6%; p=0.0315]  [T4: (IG) 20.6% vs (CG) 32.7%; ARR=11.4%; p=0.0514]  [T5: (IG) 28.4% vs (CG) 36.0%; ARR=5.2%; p=0.2496]  **Included in meta-analysis:**  (OR=0.70; 95% CI: 0.40 to 1.25)   1. IG had more significant improvement than CG at T3 and T5   Emotional function  [T3-T0: (IG) 0.50±1.0 vs (CG) 0.10±1.0; p=0.004]  [T5-T0: (IG) 0.43±1.0 vs (CG) 0.15±0.9; p=0.058]  Physical function  [T3-T0: (IG) 0.33±0.9 vs (CG) -0.01±1.0; p=0.036]  [T5-T0: (IG) 0.27±1.0 vs (CG) -0.04±1.0; p=0.016] |  |  |
| Cameron-Tucker et al. (2016) | Australia | Patients (aged 18 or above) diagnosed with COPD and 2 months after exacerbation  (n=65)  (Mean Age: 69)  **IG:** n=35,  Mean Age: 68  **CG:** n=30,  Mean Age: 70 | Community nurse | 8 weeks | **Telerehabilitation**:  **(1) Nurse telephone follow-ups**  (a) Telephone  (b) Support home-based walking plan and health behavior plan (developed pre-study from patients’ summary of SNAPPS health behavior)  (c) 2 calls a week | **(1) Written information**  (b) Personal home-based walking plan + Education information for health behaviors  **(2) Pulmonary rehabilitation (PR) program**  (a) Face-to-face  (b) Education for self-management + Gym-based supervised exercise  (c) 8 weeks, 2 hour-session weekly | (1) 8 weeks waiting time  (2) Same PR as IG | T0: baseline  T1: 8 weeks  T2: 16 weeks (follow-up) | (1) Health-related quality of life  (2) Health behaviors | (1) COPD Assessment Test (CAT)  (2) "SNAPPS" snapshot questionnaire | 1. No significant difference   [T1-T0: (IG) Median=0, IQR=6 vs (CG) Median=0, IQR=6; p=0.48]  [T2-T0: (IG) Median=0, IQR=3 vs (CG) Median=0, IQR=2; p=0.81)   1. No significant difference   [T1-T0: (IG) Median=2, IQR=6 vs (CG) Median=1, IQR=4; p=0.42]  [T2-T0: (IG) Median=0, IQR=3 vs (CG) Median=0, IQR=2; p=0.81] |  |  |
| Jolly et al. (2018) | UK | Patient (aged 18 or above) diagnosed with COPD and reported with mild dyspnea  (n=577)  (Mean Age: NA)  **IG:** n=289,  Mean Age: 70.7  **CG:** n=288,  Mean Age: 70.2 | Nurses | 12 weeks | **(1) Nurse telephone coaching**  (a) Telephone  (b) COPD self-management, lifestyle modifications, medication adherence, and management on exacerbation  (c) 35-60 mins at weeks 1, 15-20 mins at weeks 3, 7, 11 | **(1) Pre-intervention training session (For nurses)**  (b) Telephone coaching protocol  (c) 2 days  **(2) Education information**  (a) By post  (b) Self-management of COPD  (c) Weeks 16, 24 | (1) Education information by post | T0: baseline  T1: week 6  T2: week 12 | (1) Health-related quality of life → higher score poorer QoL  (2) Dyspnea  (3) Psychological morbidity  (4) Healthcare utilization | (1) Short version of the St George’s Respiratory Questionnaire (SGRQ-C)  (2) MRC Dyspnea Scale  (3) Hospital Anxiety and Depression Scale  (4) Respiratory hospital admissions | 1. No significant difference   [T1: (IG) 28.6±17.1 vs (CG) 30.5±16.7; p=0.76]  [T2: (IG) 27.9±15.7 vs (CG) 30.9±17.0; p=0.23]  **Included in meta-analysis:**  (SMD=0.18; 95% CI: 0.00 to 0.36)   1. No significant difference → OR>1 favors IG   [T1: OR=0.8; 95% CI: 0.6 to 1.2; p=0.39]  [T2: OR=1.1; 95% CI: 0.7 to 1.5; p=0.79]   1. No significant difference   Anxiety  [T1: (IG) 3.8±3.8 vs (CG) 4.5±4.0; p=0.21]  [T2: (IG) 4.0±3.8 vs (CG) 4.7±4.0; p=0.81]  **Included in meta-analysis:**  (SMD=0.18; 95% CI: 0.02 to 0.34)  Depression  [T1: (IG) 3.1±3.0 vs (CG) 3.5±3.1; p=0.21]  [T2: (IG) 3.3±3.3 vs (CG) 3.8±3.4; p=0.63]  **Included in meta-analysis:**  (SMD=0.15; 95% CI: -0.03 to 0.33)   1. No significant difference   [T1: (IG) 0.02±0.1 vs (CG) 0.03±0.2; p=0.38]  [T2: (IG) 0.01±0.1 vs (CG) 0.01±0.1; p=0.57] |  |  |
| Lavesen et al. (2016) | Denmark | Patients diagnosed with COPD  (n=213)  (Mean Age: NA)  **IG:** n=119,  Mean Age: 69.72  **CG:** n=94,  Mean Age: 70.90 | Nurses | 30 days | **(1) Nurse telephone follow-ups**  (a) Telephone  (b) Patient education on signs of exacerbations and COPD management (guided by a semi-structured manual)  (c) Total: 2 calls between 2 days and 30 days after discharge + additional calls if needed, Average: 11 mins | NA | (1) Usual care | T0: baseline  T1: 30 days  T2: 84 days (follow-up) | (1) Hospital readmission  (2) Disease management (patient self-assessment) | (1) Readmission rate  (2) Self-produced questionnaire using questions in Short Form-12 questionnaire | 1. No significant difference   [T1: (IG) 33% vs (CG) 34%; p=0.84]  [T2: (IG) 32% vs (CG) 27%; p=0.66]  **Included in meta-analysis:**  (OR=1.24; 95% CI: 0.64 to 2.38)   1. Disease management (only at T1­)   Sufficient knowledge  No significant difference (p=0.109)  Managing dyspnea  IG significantly higher than CG (p=0.002)  React on signs of exacerbations  IG significantly higher than CG (p<0.001)  Manage COPD symptoms in daily life  IG significantly higher than CG (p=0.028)  Communicate with health professional  IG significantly higher than CG (p<0.001) |  |  |
| Lee et al. (2015) | Korea | Patient (aged 40-80) diagnosed with COPD  (n=151)  Mean Age: 66.1  **IG:** n=78,  Mean Age: 66.7  **CG:** n=73,  Mean Age: 65.4 | Nurses | 6 months | **Problem Solving Theory (PST) Program**  **(1) Nurse telephone counseling**  (a) Telephone  (b) PST: develop behavioral strategies and actions plan on COPD self-care and lifestyle modifications in 1^st^ session + Positive verbal reinforcement in other sessions  (c) 2 calls per week, Total: 12 calls, 1^st^ session: 60 mins, Other sessions: max 30 mins | **(1) Pre-intervention training (For nurses)**  (b) Problem-solving counseling  (c) 16 hours  **(2) Education booklet**  (b) COPD self-management | Usual care | T0: baseline  T1: 6 months | (1) Depressive symptoms | (1) The Center for Epidemiologic Studies-Depression Scale (CES-D)  🡪 lower score, less depression | **All subjects (N=151)**   1. No significant difference   [T1: (IG) 15.9±8.0 vs (CG) 17.2±8.0; p=0.283]  **Included in meta-analysis:**  (SMD=0.16; 95% CI: -0.16 to 0.48)  **Subgroup: CES-D≥24 (N=25)**   1. No significant difference in between-group comparison, but IG significantly improved   [(IG) T0: 30.7±6.3 vs T1: 23.9±9.6; p=0.041]  [(CG) T0: 29.4±8.7 vs T1: 27.0±7.9; p=0.354]  [T1: (IG) 23.9±9.6 vs (CG) 27.0±7.9; p=0.303] |  |  |
| Prabhakaran & Wei (2019) | Singapore | Patients (aged 21 or above) with a primary diagnosis of asthma  (n=424)  (Mean Age: NA)  **IG:** n=212,  Mean Age: 37.1  **CG:** n=212,  Mean Age: 40.5 | Asthma nurses | 5 weeks | **eCARE Home Monitoring Information Technology System:**  **(1) Telemonitoring**  (a) SMS questions + SMS/email alerts  (b) Monitor asthma symptoms + SMS/email alerts to nurses for abnormal symptoms  (c) Daily for weeks 1-2, weekly for weeks 3-5  **(2) Nurse telephone follow-ups**  (a) Telephone  (b) Monitor patients with abnormal symptoms + Referral to outpatient clinic or GP or polyclinic if no improvement  (c) NA | **(1) Education session**  (a) Face-to-face  (b) Individualized asthma education  (c) Once, Average 30 mins | (1) Same education session as IG  (2) Usual outpatient follow-up | T0: baseline  T1: 5 weeks  T2: 3 months (follow-up) | (1) Number of asthma-related hospital admissions | (1) Number of asthma-related hospital admissions | 1. No significant difference   [T1: (IG) n=8, 4.8% vs (CG) n=7, 4.1%; p=0.797]  [T2: (IG) n=12, 7.5% vs (CG) n=8, 4.9%; p=0.364]  **Included in meta-analysis:**  (OR=1.53; 95% CI: 0.61 to 3.82) |  |  |
| Ringbæk et al. (2015) | Denmark | Patients with stable severe/very severe COPD at high risk of exacerbations and hospital admissions  (n=281)  (Mean Age: NA)  **IG:** n=141,  Mean Age: 69.8  **CG:** n=140, Mean Age: 69.4 | Respiratory Nurses | 6 months | **(1) Telemonitoring + Nurse video consultation**  (a) Tablet with web camera, microphone, measurement equipment (spirometer, pulse oximeter, bathroom scale)  (b) Data transferred to nurse call center and a database (accessed by multidiscipline) + system to categorize, prioritize the patient’s condition  (c) Measurement by patient alone: three times a week for the 1^st^ 4 weeks, once a week afterwards  Measurement by nurse video consultation with spirometry: Once a week for the 1^st^ 4 weeks, once a month afterwards  **(2) Nurse Follow-ups**  (a) Video consultation/ email/ telephone  (b) Follow up on alarming symptoms  (c) If needed | (1) No regular scheduled visits to outpatient clinics | Respiratory nurses follow-up at home/ outpatient clinic | T0: baseline  T1: 6 months | (1) Hospital admissions for COPD | (1) Hospital admissions for COPD | 1. No significant difference   Number of hospital admissions for COPD  [T1: (IG) Mean: 0.55 vs (CG) Mean: 0.54; p=0.74]  % of patients with at least one hospitalization for COPD  [T1: (IG) 29.1% vs (CG) 31.4%; p=0.67]  Time to first hospital admission for COPD exacerbation  No significant difference  **Included in meta-analysis:**  (OR=0.89; 95% CI: 0.54 to 1.49) |  |  |
| Soriano et al. (2018) | Spain | Patients (aged 50-90) diagnosed with COPD and treated with chronic home oxygen therapy  (n=229)  (Mean Age: NA)  **IG:** n=115,  Mean Age: 71.5  **CG:** n=114,  Mean Age: 71.3 | Nurses and Pulmonologists | 12 months | **PROMETE II**  **(1) Telemonitoring**  (a) Measurement equipment (pulse oximeter, blood pressure gauge, spirometer, respiratory rate and oxygen therapy compliance monitor)  (b) Receive data from patients (to secure server) + System will generate alerts for COPD exacerbation (traffic light system)  (c) Measurement by patient daily (at rest, after taking medication, and with oxygen therapy)  **(2) Nurse Follow-ups**  (a) Telephone (patients) + Email (pulmonologists)  (b) Confirm patient’s alert with a standardized clinical questionnaire + refer patient to a pulmonologist by email  (c) If needed  **(3) Pulmonologist Follow-ups**  (a) Telephone  (b) Provide medical suggestions for patients regarding COPD exacerbation  (c) If needed, average 5 mins | **(1) Initial nurse home visit**  (a) Face-to-face  (b) Perform initial measurement under supervision + titrate alert configuration level  (c) Once  **(2) Instruction sheet**  (b) Instruction on how to measure physiological parameters correctly | Usual care | T0: baseline  T1: 12 months | (1) Number of hospitalization/Emergency department visits due to COPD exacerbation  (2) Number of exacerbations  (3) COPD symptoms  (4) Anxiety  (5) Depression  (6) Quality of life | (1) Number of hospitalizations/Emergency department visits due to COPD exacerbation  (2) Number of exacerbations  (3) COPD Assessment Test (CAT)  (4) Goldberg Anxiety Subscale  (5) Goldberg Depression Subscale  (6) EuroQOL | 1. No significant difference   Number of hospital admissions  [T1: (IG) n=69, 60.0% vs (CG) n=61, 53.5%; p=0.321]  **Included in meta-analysis:**  (OR=1.30; 95% CI: 0.77 to 2.20)  Mean duration of hospitalizations (days)  [T1: (IG) 18.9±16.05 vs (CG) 22.4±19.52; p=0.308]  Number of ICU admissions  [T1: (IG) n=3, 2.6% vs (CG) n=3, 2.6%; p=0.991]  Number of days in ICU  [T1: (IG) 6.0±4.6 vs (CG) 13.3±11.1; p=0.349]   1. No significant difference   [T1: (IG) 1.1±1.13 vs (CG) 0.9±1.04; p=0.181]   1. No significant difference   [T1: (IG) 21.5±5.6 vs (CG) 21.4±6.1; p=0.855]   1. No significant difference   [T1: (IG) 0.9±1.9 vs (CG) 1.0±2.0; p=0.911]  **Included in meta-analysis:**  (SMD=0.05; 95% CI: -0.21 to 0.31)   1. No significant difference   [T1: (IG) 1.8±2.21 vs (CG) 2.2±2.64; p=0.316]  **Included in meta-analysis:**  (SMD=0.16; 95% CI: -0.10 to 0.42)   1. No significant difference   [T1: (IG) 0.80±0.2 vs (CG) 0.79±0.2; p=0.895]  **Included in meta-analysis:**  (SMD=0.05; 95% CI: -0.21 to 0.31) |  |  |
| Tupper et al. (2018) | Denmark | Patients with stable severe/very severe COPD at high risk of exacerbations and hospital admissions  (n=281)  (Mean Age: NA)  **IG:** n=141,  Mean Age: 69.8  **CG:** n=140,  Mean Age: 69.4 | Respiratory Nurses | 6 months | **(1) Telemonitoring + Nurse video consultation**  (a) Tablet with web camera, microphone, measurement equipment (spirometer, pulse oximeter, bathroom scale)  (b) Data transferred to nurse call center and a database + system to categorize, prioritize the patient’s condition (coded green, yellow, red)  (c) Measurement by patient alone: three times a week for the 1^st^ 4 weeks, once a week afterwards  Measurement by nurse video consultation with spirometry: Once a week for the 1^st^ 4 weeks, once a month afterwards  **(2) Nurse Follow-ups**  (a) Video consultation/ email/ telephone  (b) Follow-up alarming symptoms  (c) If needed | (1) No regular scheduled visits to outpatient clinics | Usual outpatient pulmonary rehabilitation visits once/twice a year | T0: baseline  T1: 6 months | (1) Health-related quality of life | (1) 15D questionnaire  (2) COPD Assessment Test (CAT) | 1. IG had a significantly higher score than CG   [T1: (IG) 0.761±0.107 vs (CG) 0.728±0.121; p=0.02]  [(IG) T1-T0: 0.016; 95% CI: 0.002, 0.031; p=0.03]  [(CG) T1-T0: -0.003; 95% CI: -0.018, 0.012; p=0.68]  **Included in meta-analysis:**  (SMD=0.29; 95% CI: 0.04 to 0.54)   1. No significant difference   [T1: (IG) 5.6±6.9 vs (CG) 27.5±7.5; p=0.36]  [(IG) T1-T0: -0.76; 95% CI: -1.68, 0.15; p=0.101]  [(CG) T1-T0: -0.73; 95% CI: -1.74, 0.28; p=0.154] |  |  |

4. Diabetes

| **Author**  **(Year of publication)** | **Study Location** | **Study Population** | **Provider** | **Duration** | **Intervention Group (IG)** | | **Control Group (CG)** | **Data Collection Timepoint** | **Outcome Variables** | **Outcome Measures** | **Result** |
| --- | --- | --- | --- | --- | --- | --- | --- | --- | --- | --- | --- |
|  |  |  |  |  | **Technological**  **(a) Delivery mode**  **(b) Content**  **(c) Frequency/Duration** | **Non-technological**  **(a) Delivery mode**  **(b) Content**  **(c) Frequency/Duration** |  |  |  |  |  |
| Fernandes et al. (2016) | Brazil | Patients (aged 30-80) diagnosed with Type 2 DM  (n=210)  (Mean Age: NA)  **IG:** n=104,  Mean Age: NA  **CG:** n=106,  Mean Age: NA | Nurses | 6 months | **(1) Telephone intervention**  (a) Telephone  (b) Encouragement given to achieve individualized goals on DM self-care (according to a previously established protocol)  (c) Monthly, Total: 6 telephone calls, Average: 14.0 mins | NA | **(1) Usual care**  (b) Conventional monitoring in clinic  **(2) Educational booklet**  (b) DM + DM self-care (preventing complications, lifestyle modifications) | T0: baseline  T1: 3 months  T2: 6 months | (1) Self-care | (1) Diabetes Self-care Questionnaire | 1. IG had significantly greater improvement than CG in T2   [T1: (IG) 5.20±0.94 vs (CG) 4.34±1.10]  [T2: (IG) 5.53±0.80 vs (CG) 4.10±1.18]  [T2-T1: (IG) 1.97±0.96 vs (CG) 0.57±1.53; p=0.000]  **Included in meta-analysis:**  (SMD=1.41; 95% CI: 1.11 to 1.71) |
| Hansen et al. (2017) | Denmark | Patients (aged 30-75) diagnosed with Type 2 DM and participated in a diabetes program without reaching their goal prior recruitment  (HbA1c > 7.5%)  (BMI > 25)  (n=165)  (Mean Age: 58)  **IG:** n=83,  Mean Age: 57.8  **CG:** n=82,  Mean Age: 58.3 | Nurses | 8 months | **(1) Videoconferencing**  (a) Tablet  (b) Empowerment on DM self-management and consequences of hypoglycemia  (c) Monthly  **(2) Telemonitoring**  (a) Tablet + measurement tools  (b) Patients regularly upload measurements of blood glucose, blood pressure, and weight  (c)  - Blood pressure: once a week  - Weight: once a week  - Blood glucose: twice a day 🡪 (on insulin) daily; (no insulin) weekly | (**1) Pre-intervention education program (For nurses)**  (b) Type 2 DM + management of hypoglycemia  **(2) Usual care**  (b) Clinic visit every 3-6 months) | Usual care (clinic visit every 3-6 months) | T0: baseline  T1: 4 months  T2: 8 months  T3: 14 months (follow-up) | (1) HbA1c  (2) Quality of life | (1) HbA1c  (2) SF-36 | 1. IG had a more significant reduction than CG in T2, but no significant difference in T3   [T2-T0: (IG) -0.69; p<0.000001 vs (CG) -0.18; p=0.22]  [T3-T2: (IG) +0.18; p=NA vs (CG) -0.12; p=NA]  (2) No changes |
| Hemmati Maslakpak et al. (2017) | Iran | Patients (aged 18-55) diagnosed with Type 2 DM without using insulin  (n=90)  (Mean Age: NA)  **IG:**  Arm 1: n=30, Mean Age: 49.9  Arm 2: n=30, Mean Age: 49.46  **CG:** n=30,  Mean Age: 50.6 | Nurses | 3 months | **Arm 2: Telephone-based Family-orientated Education**  **(1) Telephone education session**  (a) Telephone (patient and family called separately)  (b) DM self-management, lifestyle modifications, Foot ulcer prevention, medication adherence + Answer patients’ queries  (c) Twice a week in the first 1^st^ and 2^nd^ months, Once a week in the 3^rd^ month, 15-30 mins | **Arm 1: Face-to-face Family-orientated Education**  **(1) Face-to-face education session**  (a) Face-to-face  (b) DM self-management, lifestyle modification, Foot ulcer prevention, medication adherence + Answer patients’ enquiry  (c) Twice a week in 1^st^ month, Once a week in 2^nd^ and 3^rd^ months, 20-30 minutes | **Usual education**  (1) Monthly training session  (2) Education materials | T0: baseline  T1: 3 months | (1) Self-care  (2) HbA1c  (3) Fasting blood sugar | (1) Persian version of SDSCA questionnaire  (2) HbA1c  (3) Fasting blood sugar | 1. Arm 1 had a higher score than Arm 2 and CG in T1   [T1: (Arm 1) 100.82±14.56 vs (Arm 2) 92.93±11.09 vs (CG) 49.46±16.35; p=0.001]  [T1: (Arm 1 vs CG) p=0.0001; (Arm 2 vs CG) p=0.0001; (Arm 1 vs Arm 2) p=0.011]  **Included in meta-analysis (Arm 2 vs CG)**  (SMD=3.07; 95% CI: 2.31 to 3.83)   1. No significant difference   [T1: (Arm 1 vs CG) p=0.344; (Arm 2 vs CG) p=0.236; (Arm 1 vs Arm 2) p=0.971)   1. No significant difference   [T1: (Arm 1 vs CG) p=0.384; (Arm 2 vs CG) p=0.766; (Arm 1 vs Arm 2) p=0.804] |
| Kim & Utz (2019) | Korea | Patients diagnosed with Type 2 DM (HbA1c > 7.0%)  (n=155)  (Mean Age: NA)  **IG:**  SM-HL: n=52, Mean Age: 46.00  TEL-HL: n=51, Mean Age: 52.00  **CG:** n=52,  Mean Age: 56.00 | Nurses | 8 weeks | **SM-HL: Social Media-Based, Health Literacy Sensitive Diabetes Management Intervention**  **(1) Social media support**  (a) Smartphone, Apps: Mastering Diabetes  (b) Action planning + Answering enquiries + Upload education materials (e.g., video, short text) + Discussion forum  (c) Weekly  **TEL-HL: Telephone-Based, Health Literacy Sensitive Diabetes Management Intervention**  **(1) Telephone support**  (a) Telephone  (b) Action planning + Answering enquiries  (c) Weekly | **(1) Pre-intervention training (For nurse)**  (b) Teach-back method, Action-planning strategies  **(2) Initial education session**  (a) Face-to-face  (b) General information on diabetes self-management  (c) Once  **(2) Educational brochure**  (b) Easy-to-read diabetes education brochure | **Usual Education**  **(1) Initial education session**  🡪 Same as IG  **(2) Educational brochure**  (b) Conventional diabetes education brochure | T0: baseline  T1: 9 weeks  T2: 12 weeks (HbA1c only) | (1) Self-care behavior  (2) HbA1c | (1) Revised Korean version of Summary of Diabetes Self-care Activities Measure (SDSCA)  (2) HbA1c | 1. No significant changes   [T1: (SM-HL) 4.62±0.96 vs (TEL-HL) 5.04±0.79 vs (CG) 4.54±1.05; p=0.581]  **Included in meta-analysis (TEL-HL vs CG)**  (SMD=0.53; 95% CI: 0.14 to 0.93)   1. No significant changes   [T1: (SM-HL) 7.21±1.42 vs (TEL-HL) 7.31±1.35 vs (CG) 6.83±0.96; p=0.840]  [T2: (SM-HL) 6.98±1.36 vs (TEL-HL) 7.40±1.90 vs (CG) 6.74±1.07; p=0.827]  **Included in meta-analysis (TEL-HL vs CG)**  (MD=-0.66; 95% CI: -1.26 to -0.06) |
| Kim et al. (2021) | Korea | Patients (aged 19 or above) diagnosed with Type DM and taking DM medications or insulin for more than 6 months  (HbA1c > 6.5%)  (n=68)  (Mean Age: 55.18)  **IG:** n=32,  Mean Age: NA  **CG:** n=36,  Mean Age: NA | Nurses | 8 weeks | **(1) Smartphone application**  (a) Smartphone + Apps: Doctor Diary  (b) Patients record their blood sugar, diet, medication, daily steps (Tele-monitoring: revised by nurses daily) + Chat function  (c) 3 times a week (chat function)  **(2) Phone counseling**  (a) Telephone  (b) Answer enquiries on diabetes self-management (structured by a manual)  (c) Weekly, 10 mins per session | **(1) Education session**  (a) Face-to-face  (b) Diabetes self-management (validated educational materials developed from research and guidelines) + App training  (c) Once, 60 mins + 30 mins (training)  **(2) Educational booklet**  (b) Diabetes self-management | **Usual care**  **(1) Educational booklet**  (b) “Diabetes Management Guide”  (c) Routinely given to patient | T0: baseline  T1: 8 weeks | (1) HbA1c  (2) Fasting blood sugar  (3) Diabetes self-management behaviors | (1) HbA1c  (2) Fasting blood sugar  (3) Revised Summary of Diabetes Self-Care Activities Measure Scale (SDSCA) | 1. IG had significantly lower HbA1c than CG   [T1: (IG) 6.88±1.37 vs (CG) 7.39±1.40]  [T1-T0: (IG) -1.01±1.29 vs (CG) -0.09±0.70; p<0.001]  **Included in meta-analysis**  (MD=0.51; 95% CI: -0.15 to 1.17)   1. IG had significantly decreased fasting blood sugar   [(IG) T0: 154.63±71.61 vs T1: 124.02±17.79; Mean difference: -30.61±62.01; p=0.009]   1. IG had more significant improvement in self-care than CG   [T1: (IG) 84.25±14.63 vs (CG) 75.17±17.93]  [T1-T0: (IG) 14.75±12.80 vs (CG) 4.72±12.81; p=0.002]  **Included in meta-analysis**  (SMD=0.55; 95% CI: 0.06 to 1.03) |
| Li et al. (2017) | Canada | Patients (aged 18 or above) diagnosed with Type 2 DM  (HbA1c ≥ 8%)  (n=134)  (Mean Age: 57.43)  **IG:** n=70,  Mean Age: 56.69  **CG:** n=64,  Mean Age: 58.23 | Nurse case manager (NCM) | 6 months | **(1) Monitoring and Medication Titration**  (a) Telephone or email  (b) Patients communicate blood glucose readings to NCM + Insulin adjustment by NCM (following algorithm) + Medication adjustment by NCM communicating with endocrinologist  (c) NA  **(2) NCM follow-up**  (a) Telephone or email  (b) DM education + Dose adjustment support + Psychological support  (c) Agreed upon with patients and NCM, Daily (for dose adjustment)  **(3) Referral**  (a) NA  (b) Referral to endocrinologists, family physicians, or other clinics in the community  (c) As required | **(1) Initial consultation with NCM**  (a) Face-to-face  (b) DM-related information + Setting goal  (c) Once  **(2) Educational material**  (b) Written/Picture-based diabetes-related information | **Standard care:** clinic visit | T0: baseline  T1: 6 months | (1) HbA1c level  (2) Self-management behavior  (3) Depressive symptom severity | (1) HbA1c level  (2) Revised Summary of Diabetes Self-Care Activities Measure  (3) 9-item PRIME-MD Patient Health Questionnaire (PHQ-9) | 1. Both groups had significantly reduced HbA1c, but IG had greater improvement   [T1: (IG) 7.72±1.43 vs (CG) 8.46±2.29]  [T1-T0: (IG) -2.72±2.54; p<0.01]  [T1-T0: (CG) -2.06±2.58; p<0.01]  [T1-T0: (IG) vs (CG); p=0.027]  **Included in meta-analysis**  (MD=0.74; 95% CI: 0.09 to 1.39)   1. IG had significant improvement   Fruit and vegetable intake  [T1-T0: (IG) 1.02±2.86 vs (CG) -0.26±2.92]  [Adjusted difference between groups: 1.112; 95% CI: (0.274, 1.950); p=0.010]  Fat intake  [T1-T0: (IG) 0.08±1.9 vs (CG) 0.07±2.94]  [Adjusted difference between groups: -0.761; 95% CI: (-1.541, 0.019); p=0.056]  Physical exercise  [T1-T0: (IG) 0.94±3.5 vs (CG) -0.17±2.83]  [Adjusted difference between groups: 1.277; 95% CI: (0.359, 2.195); p=0.007]  Specific exercise  [T1-T0: (IG) 0.77±3.19 vs (CG) -0.29±2.97]  [Adjusted difference between groups: 1.319; 95% CI: (0.418, 2.220); p=0.005]  Foot exam  [T1-T0: (IG) 2.02±3.56 vs (CG) 0.62±3.44]  [Adjusted difference between groups: 1.006; 95% CI: (0.004, 2.008); p=0.049]  Blood glucose monitoring  [T1-T0: (IG) 0.85±2.79 vs (CG) 0.90±3.12]  [Adjusted difference between groups: -0.259; 95% CI: (-1.069, 0.551); p=0.528]   1. IG had significantly less depression   [T1-T0: (IG) -1.82±4.48 vs (CG) 1.09±4.42]  [Adjusted difference between groups: -2.954; 95% CI: (-4.381, -1.527); p<0.001] |
| Odnoletkova et al. (2016) | Belgium | Patients (aged 18-75) diagnosed with Type DM and taking glucose-lowering oral medications or injection  (n=574)  (Mean Age: NA)  **IG:** n=287,  Mean Age: 63.8  **CG:** n=287,  Mean Age: 62.4 | Diabetes nurses | 6 months | **COACH Programme:**  **(1) Nurse telephone sessions**  (a) Telephone  (b) Motivational interviewing to enhance the lifestyle modifications and medication adherence of patients  (c) Mean interval: 5 weeks, Mean duration: 30 mins | **(1) Pre-intervention training (For nurses)**  (b) “Guidelines for the management of Type 2 diabetes, motivational interviewing techniques and software program use”  (c) 5 days  **(2) Welcome package**  (b) Nutrition guide + measurement tools (waist circumference meter, BMI calculator, blood glucose monitoring set)  (2) Educational material with DVD | (1) Usual care: follow-up with GP  (2) Educational material with DVD | T0: baseline  T1: 6 months  T2: 18 months | (1) HbA1c | (1) HbA1c | 1. IG had significantly reduced HbA1c and was below the recommended target (7%)   [T1: (IG) N=252, 6.8±0.9 vs (CG) N=260, 7.0±1.1; p=0.003]  [T2: (IG) N=240, 6.9±1.0 vs (CG) N=246, 7.0±1.1; p=0.046]  **Included in meta-analysis**  (MD=0.10; 95% CI: -0.09 to 0.29) |
| Sherifali et al. (2021) | Canada | Patients (aged 18 or above) diagnosed with Type 2 DM  (HbA1c > 7.5%)  (n=365)  (Mean Age: NA)  **IG:** n=188,  Mean Age: 56.82  **CG:** n=177,  Mean Age: 59.05 | Nurses and certified diabetes educators | 12 months | **(1) Telephone counseling**  (a) Telephone  (b) Case management, DM self-management education, psychological support  (c) Weekly in the 1^st^ 6 months, Monthly in the last 6 months | **(1) Usual DM education**  (b) Community resources + accelerometer to monitor activity level | **(1) Usual DM education**  (b) Community resources + accelerometer to monitor activity level | T0: baseline  T1: 6 months  T2: 12 months | (1) HbA1c  (2) Quality of life  (3) Self-care  (4) Hospital admissions | (1) HbA1c  (2) 19-item Audit of Diabetes-Dependent Quality of Life (ADDQoL-19) scale  (3) Summary of Diabetes Self-Care Activities (SDSCA) scale  (4) Hospital admissions | 1. IG had a more significant reduction in HbA1c than CG   [T2-T0: (IG) -1.78; 95% CI: 2.00 to -1.57; p<0.005]  [T2-T0: (CG) -1.29; 95% CI: -1.52 to -1.07; p<0.005]  [T2: (IG-CG) -0.48; 95% CI: -0.79 to -0.17; p<0.005]   1. IG had more significant improvement than CG   [T2-T0: (IG) 0.208; 95% CI: 0.044 to 0.371; p>0.005]  [T2-T0: (CG) -0.012; 95% CI: -0.181 to 0.157; p>0.005]  [T2: (IG-CG) 0.277; 95% CI: 0.039 to 0.515; p=0.02]   1. No significant difference   [T2-T0: (IG) 0.730; 95% CI: 0.536 to 0.923; p<0.05]  [T2-T0: (CG) -0.586; 95% CI: 0.385 to 0.1788; p<0.05]  [T2: (IG-CG) 0.143; 95% CI: -0.135 to 0.422; p>0.05]   1. No significant difference   [(IG) n=24, 12.77% vs CG: n=27, 15.25%) |
| Tan et al. (2020) | Singapore | Patients (aged 50 or above) diagnosed with Type 2 DM  (HbA1c > 8%)  (n=122)  (Mean Age: NA)  **IG:** n=57,  Mean Age: 61.50  **CG:** n=65,  Mean Age: 62.75 | Nurses | 8 weeks | **Diabetes Self-Efficacy Enhancing Program (DSEEP)**  **(1) Nurse telephone follow-up**  (a) Telephone  (b) Reinforce DM self-management skills + Answer enquiries and offer advice on dealing with difficulties  (c) Every 2 weeks, Total: 3 calls | **(1) Educational materials**  (b) DM guidebook + digital videodisc  **(2) Training workshop**  (a) Face-to-face  (b) DM self-management, insulin injection techniques  (c) Once, 3 hrs  **(3) Usual follow-up in polyclinic** | **(1) Usual care**  (b) Follow-up consultation in polyclinic | T0: baseline  T1: 8 weeks | (1) Self-care  (2) Health-related quality of life  (3) HbA1c  (4) Unplanned health care service usage | (1) Revised Summary of Diabetes Self-Care Activities (RSDSA) scale  (2) Audit of Diabetes-Dependent Quality of Life (ADDQoL)  (3) HbA1c  (4) Unplanned diabetes-related medical consultations | 1. IG had a more significant increase than CG   [T1: (IG) N=56, 4.81±1.11 vs (CG) N=57, 3.92±1.35]  [T1-T0: (IG) N=56, 1.18; p<0.001]  [T1-T0: (CG) N=57, 0.28; p=0.05]  [Interaction effect: F(1, 111)=18.11; p<0.001]  **Included in meta-analysis**  (SMD=0.71; 95% CI: 0.33 to 1.10)   1. No significant difference   General  [T1: (IG) N=56, -0.05±0.49 vs (CG) N=57, -0.36±0.61]  [T1-T0: (IG) N=56, 0.27; p=0.009]  [T1-T0: (CG) N=57, 0.10; p=0.40]  [Interaction effect: F(1, 111)=1.37; p=0.24]  Diabetes-specific  [T1: (IG) N=56, -4.06±2.12 vs (CG) N=57, -3.30±2.18]  [T1-T0: (IG) N=56, -0.27; p=0.17]  [T1-T0: (CG) N=57, -0.42; p=0.04]  [Interaction effect: F(1, 111)=6.11; p=0.05]   1. IG had a more significant reduction in HbA1c than CG   [T1: (IG) N=56, 8.66±1.24 vs (CG) N=57, 9.04±1.54]  [T1-T0: (IG) N=56, -1.24; p<0.001]  [T1-T0: (CG) N=57, -0.58; p=0.001]  [Interaction effect: F(1, 111)=6.08; p=0.01]  **Included in meta-analysis**  (MD=0.38; 95% CI: -0.14 to 0.90)   1. IG had a more significant reduction in hospitalizations than CG   [T1: (IG) -7.14% vs (CG) -3.51%] |

5. Cancer

| **Author**  **(Year of publication)** | **Study Location** | **Study Population** | **Provider** | **Duration** | **Intervention Group (IG)** | | **Control Group (CG)** | **Data Collection Timepoint** | **Outcome Variables** | **Outcome Measures** | **Result** |
| --- | --- | --- | --- | --- | --- | --- | --- | --- | --- | --- | --- |
|  |  |  |  |  | **Technological**  **(a) Delivery mode**  **(b) Content**  **(c) Frequency/Duration** | **Non-technological**  **(a) Delivery mode**  **(b) Content**  **(c) Frequency/Duration** |  |  |  |  |  |
| Beaver et al. (2017) | UK | Patients diagnosed with stage-I endometrial cancer and completed primary treatment  (n=259)  (Median Age: 65)  **IG:** n=129, Median Age: 66  **CG:** n=130, Median Age: 64 | Gynecology oncology nurse specialists | 12 months | **(1) Nurse Telephone Follow-ups**  (a) Telephone  (b) Questions on physical, psychological, and social health  (c) Once, 20 mins, 3/4/6/12 months | **(1) Pre-intervention Training (For nurses)**  (b) Telephone consultation  (c) 2 sessions | **Hospital-based follow-up**  (a) Face-to-face  (b) Clinical examination + Questions on signs of recurrent disease  (c) Depends on hospital policy | T0: baseline  T1: post-intervention (3/4/6/12 months) | (1) Psychological morbidity  (2) Quality of life | (1) State Trait Anxiety Inventory (STAI)  (2) European Organization for Research and Treatment (EORTC) QLQ-C30 | 1. No significant difference   [T1: (IG) 33.0±11.0 vs (CG) 35.5±13.0]  **Included in meta-analysis**  (MD=2.50; 95% CI: -0.43 to 5.43)   1. No significant differences   [T1: (IG) 71.6±19.8 vs (CG) 73.2±21.5; Adjusted mean difference: 2.1; 95% CI: -2.0 to 6.2; p=0.31]  **Included in meta-analysis**  (SMD=-0.08; 95% CI: -0.32 to 0.17) |
| Çınar et al. (2021) | Turkey | Patients (aged 18-65) diagnosed with non-metastatic breast cancer and using adjuvant EHT  (n=64)  (Mean Age: 45.7)  **IG:** n=31,  Mean Age: 45.9  **CG:** n=33,  Mean Age: 45.5 | Nurses | 12 weeks | **(1) Mobile app-based patient education**  (a) Smartphone application  (b) Information on breast cancer, its treatment, and management of EHT + Training on relaxation techniques and guided imaginary + Symptom dairy for recording symptoms + Daily reminder + Contacting specialty nurses for questions and counseling  (c) Throughout 12 weeks  **(2) Web-based management application (for nurses)**  (a) Web-based  (b) Interface for nurses to answer patients’ questions every day and provide support within 24 hours + multi-disciplinary team for advice  (c) Throughout 12 weeks | **(1) Training session**  (a) Face-to-face  (b) Training on patient use of the app  (c) Once | Usual care | T0: baseline  T1: 12 weeks | (1) Quality of life | (1) FACT-ES Quality of Life Scale (FACT-ES QLS) | 1. IG had a significant improvement in quality of life, while CG had a significant decrease   [T1: (IG) 137.58±16.94 vs (CG) 123.7±20; p=0.004]  [T1-T0: (IG) p<0.001 vs (CG) p=0.003]  **Included in meta-analysis**  (SMD=0.74; 95% CI: 0.23 to 1.25) |
| Ghanbari et al. (2021) | Iran | Patients (aged 20-60) diagnosed with non-metastatic breast cancer with moderate-to-severe anxiety and low-to-moderate self-esteem  (n=82)  (Mean Age: 46.45)  **IG:** n=41,  Mean Age: 46.9  **CG:** n=41,  Mean Age: 46 | Psychiatric nurses | 4 weeks | **(1) Psychoeducational apps “BCSzone”**  (a) Smartphone apps  (b) Information on breast cancer, stress management, self-esteem, anger management  (c) Throughout 4 weeks  **(2) Nurse Follow-up**  (a) Telephone (WhatsApp)  (b) Provide support to patients to deal with difficulties  (c) 60 mins weekly | NA | Waitlist control | T0: baseline  T1: 4 weeks | (1) Anxiety | (1) State-Trait Anxiety Inventory (STAI) | 1. IG had a more significant improvement than CG   [T1: (IG) 90.66±13.84 vs (CG) 106.92±15.94]  [T1-T0: (IG) -13.02; 95% CI: -17.44 to -8.90; p<0.001]  [T1-T0: (CG) 0.61; 95% CI: -0.69 to 1.92; p=0.34]  **Included in meta-analysis**  (MD=16.26; 95% CI: 9.80 to 22.72) |
| Wheelock et al. (2015) | US | Patients diagnosed with stage I, II, or III breast cancer and completed acute treatment and recovered from serious side effects  (n=100)  (Mean Age: 52.85)  **IG:** n=59,  Mean Age: 54.78  **CG:** n=41,  Mean Age: 53.32 | Nurse practitioner (NP) | 18 months | **(1) SIS.NET**  (a) Online  (b) Online health questionnaires + Automatic referrals to support resources  (c) Between clinic visits  **(2) Nurse telephone follow-ups**  (a) Telephone  (b) Evaluation of health questionnaire and symptoms  (c) Between clinic visits | **(1) Standard follow-up**  (a) Face-to-face  (b) Routine follow-up in breast cancer center  (c) 3 times | Standard follow-up | T0: baseline  T1: 18 months | (1) Clinic resources use | (1) Clinic resources use | 1. No significant difference in the use of all resources  - including physician appointment, breast care center appointments, non-breast care center appointments, and medical tests |

6. Stroke

| **Author**  **(Year of publication)** | **Study Location** | **Study Population** | **Provider** | **Duration** | **Intervention Group (IG)** | | **Control Group (CG)** | **Data Collection Timepoint** | **Outcome Variables** | **Outcome Measures** | **Result** |
| --- | --- | --- | --- | --- | --- | --- | --- | --- | --- | --- | --- |
|  |  |  |  |  | **Technological**  **(a) Delivery mode**  **(b) Content**  **(c) Frequency/Duration** | **Non-technological**  **(a) Delivery mode**  **(b) Content**  **(c) Frequency/Duration** |  |  |  |  |  |
| Irewall et al. (2015)  Irewall et al. (2019)  Ögren et al. (2015) | Sweden | Patients diagnosed with stroke or Transient Ischemic Accident  (n=537)  (Mean Age: 70.8)  **IG:** n=266,  Mean Age: 71.5  **CG:** n=271,  Mean Age: 70.1 | Nurses + Physicians | 36 months | **NAILED Stroke Risk Factor Trial:**  **(1) Nurse follow-ups**  (a) Telephone  (b) Lifestyle counseling + Contact physician for medication titration if target-level not reached (BP<140/90mmHg, LDL-C<2.5)  (c) NA | (1) Measurement of BP and blood lipids within 4 weeks after medication titration | Usual care | T0: baseline  T1: 12 months  T2: 24 months  T3: 36 months | (1) SBP  (2) DBP  (3) LDL-C | (1) SBP  (2) DBP  (3) LDL-C | 1. IG had significantly decreased SBP but no significant difference in CG in T1   [T1: (IG) 131.9±15.7 vs (CG) 135.0±17.5]  [T3: (IG) 128.1 vs (CG) 134.2]   1. IG had significantly decreased DBP but no significant difference in CG   [T1: (IG) 77.3±10.3 vs (CG) 79.6±10.5]  [T3: (IG) 74.3 vs (CG) 78.8]   1. Both groups had decreased LDL-C   [T1: (IG) 2.3±0.7 vs (CG) 2.6±0.9]  [T3: (IG) 2.2 vs (CG) 2.5] |
| Kirkness et al. (2017) | US | Patients diagnosed with ischemic/hemorrhagic stroke and clinical depression  (n=100)  (Mean Age: 60)  **IG:**  Arm 1: n=37, Mean Age: 61.7  Arm 2: n=35, Mean Age: 58.5  **CG:** n=28, Mean Age: 60.7 | Advanced Practice Nurses | 8 weeks | **Living well with stroke 2 (LWWS 2)**  **Arm 1: Telephone Intervention**  (a) Telephone  (b) Education on behavioral therapy + Management on depression by problem-solving techniques + Involvement of family caregivers  (c) Total: 6 sessions | **Arms 1 & 2: Initial Orientation**  (a) Face-to-face  (b) Initial assessment and discussion on goals and participants’ expectations  (c) Once  **Arms 1 & 2: Participant manuals**  (b) Cognitive behavioral therapy  Arm 2: In-person intervention  (a) Face-to-face  (b) Same content as arm 1  (c) Total: 6 sessions | Usual care | T0: baseline  T1: 8 weeks  T2: 21 weeks  T3: 12 months | (1) Change in depressive symptoms | (1) Hamilton Rating Scale for Depression (HRSD) | 1. No significant differences between Arm 1 and Arm 2, but had a significant effect when IG and CG were compared |
| Wan et al. (2016) | China | Patients (aged above 35) diagnosed with ischemic stroke  (n=91)  (Mean Age: NA)  **IG:** n=46,  Mean Age: 59.07  **CG:** n=45,  Mean Age: 60.24 | Stroke Nurses | 3 months | **(1) Nurse Telephone Follow-ups**  (a) Telephone  (b) Stroke self-management and behavioral improvement on medication usage and lifestyle modifications (evidence-based protocol)  (c) Total: 3 sessions (1 week, 1 month, 3 months), 15-20 mins each | **(1) Education session**  (a) Face-to-face  (b) Stroke education  (c) Once | Same education session as IG | T0: baseline  T1: 3 months  T2: 6 months | (1) mRS score | (1) Modified Rankin Scale (mRS) | 1. There was significant improvement in both groups, but no significant difference between groups |

IG: Intervention group; CG: Control group

MD: Mean difference; SMD: Standardized mean difference; 95% CI: 95% confidence interval; OR: Odds ratio; ARR: Adjusted risk ratio
