# Supplementary figures and images for: Nurse-led Telehealth Intervention for Rehabilitation (Telerehabilitation) Among Community-Dwelling Patients With Chronic Diseases: Systematic Review and Meta-analysis
Source: J Med Internet Res. 2022 Nov 2;24(11):e40364. doi: 10.2196/40364 (PMC9669889; doi:10.2196/40364)

## Multimedia Appendix 3

Supplementary file 3. Overall structure of nurse follow-ups


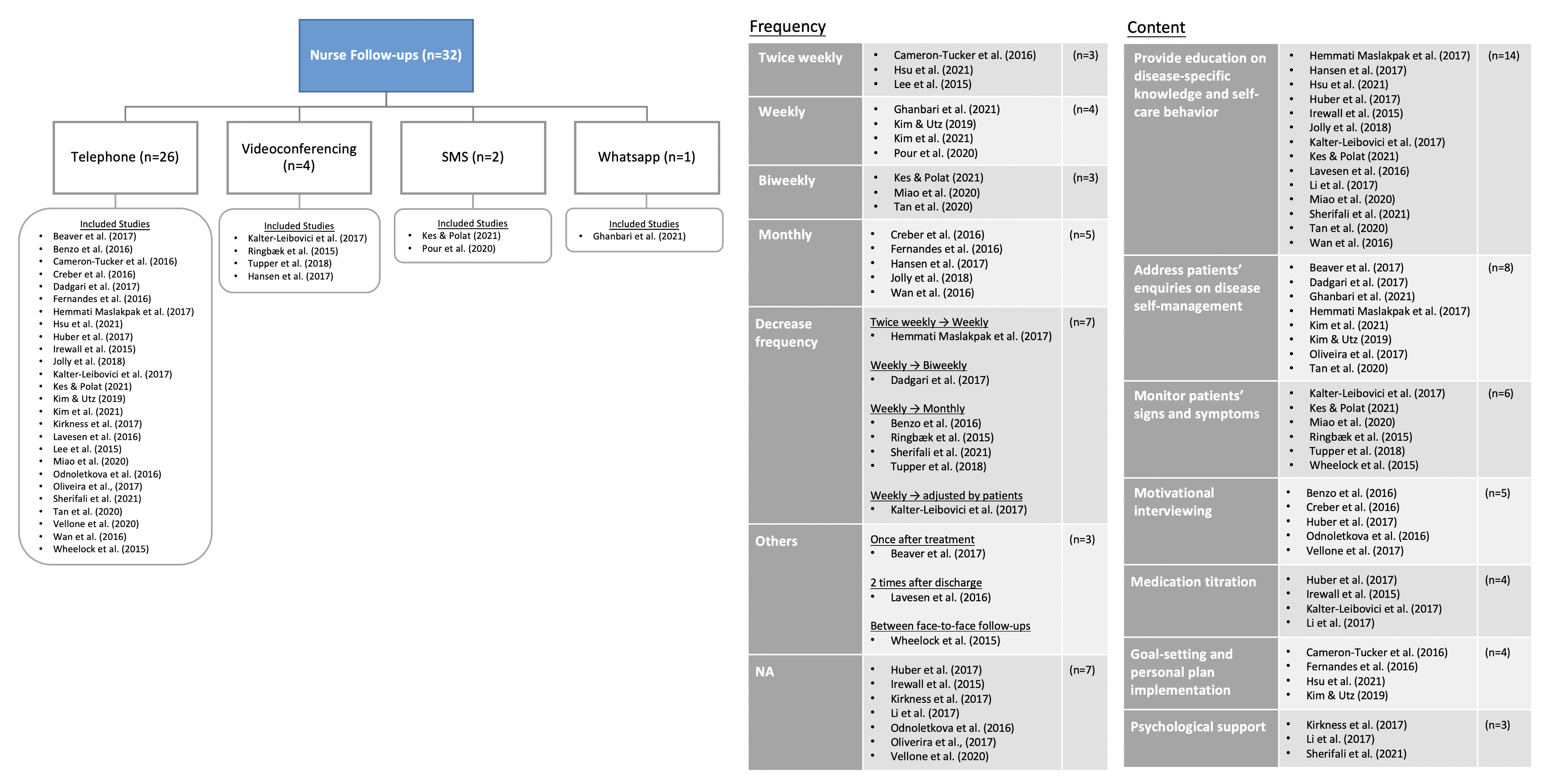

Supplement: Multimedia Appendix 3 [file jmir_v24i11e40364_app3.docx]

## Multimedia Appendix 4

Supplementary file 4. Overall structure of telemonitoring


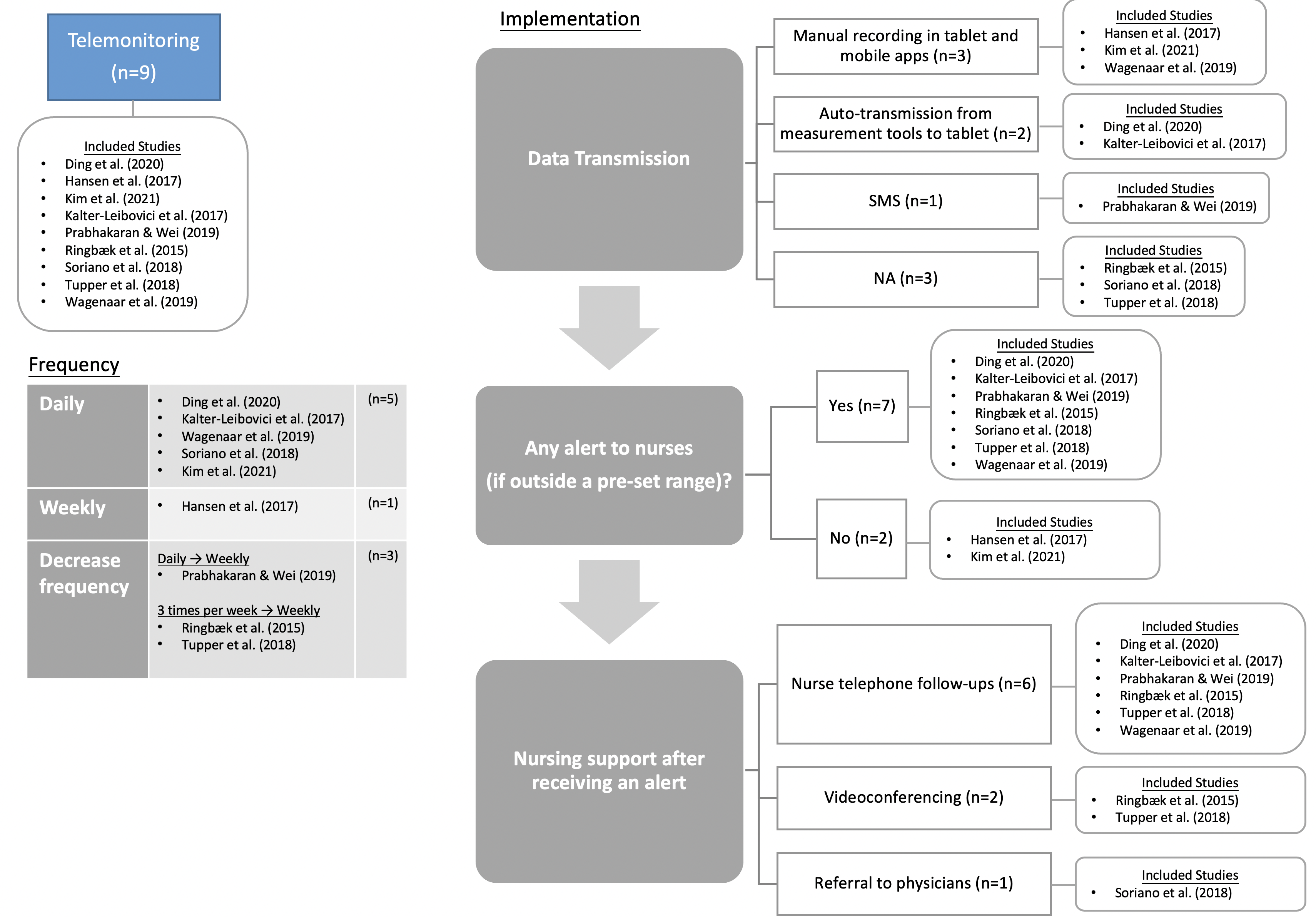

Supplement: Multimedia Appendix 4 [file jmir_v24i11e40364_app4.docx]
