# Supplementary material for: Nurse-led Telehealth Intervention for Rehabilitation (Telerehabilitation) Among Community-Dwelling Patients With Chronic Diseases: Systematic Review and Meta-analysis
Source: J Med Internet Res. 2022 Nov 2;24(11):e40364. doi: 10.2196/40364 (PMC9669889; doi:10.2196/40364)
Supplement: Multimedia Appendix 5 [file jmir_v24i11e40364_app5.docx]

## Multimedia Appendix 5

Supplementary file 5. Chronic care model

| **Study** | **Interaction between patients and nurses** | **Self-management support** | **Delivery system design (i.e. regular FU)** | **Decision support** | **Clinical information system** | **Community resources** |  |
| --- | --- | --- | --- | --- | --- | --- | --- |
| **1. Hypertension** | | | | | | |  |
| Dadgari et al. (2017) | Yes | Yes | Yes | No | No | No |  |
| Kes & Polat (2021) | Yes | Yes | Yes | No | No | No |  |
| Miao et al. (2020) | Yes | Yes | Yes | Yes | No | Yes |  |
| Pour et al. (2020) | Yes | Yes | Yes | No | No | No |  |
| **2. Cardiac Diseases** | | | | | | |  |
| Creber et al. (2016) | Yes | Yes | Yes | Yes | No | No |  |
| Ding et al. (2020) | Yes | Yes | Yes | Yes | Yes | No |  |
| Hsu et al. (2021) | Yes | Yes | Yes | No | No | No |  |
| Huber et al. (2017)  Henriksson et al. (2021) | Yes | Yes | Yes | Yes | No | No |  |
| Kalter-Leibovici et al. (2017) | Yes | Yes | Yes | Yes | Yes | Yes |  |
| Oliveira et al. (2017) | Yes | Yes | Yes | Yes | No | No |  |
| Peng et al. (2018) | Yes | Yes | Yes | Yes | No | Yes |  |
| Vellone et al. (2020) | Yes | Yes | Yes | No | No | No |  |
| Wagenaar et al. (2019) | Yes | Yes | Yes | Yes | Yes | No |  |
| **3. Chronic Respiratory Diseases** | | | | | | |  |
| Benzo et al. (2016)  Benzo & McEvoy (2019) | Yes | Yes | Yes | No | No | No |  |
| Cameron-Tucker et al. (2016) | Yes | Yes | Yes | Yes | No | No |  |
| Jolly et al. (2018) | Yes | Yes | Yes | Yes | No | No |  |
| Lavesen et al. (2016) | Yes | Yes | Yes | Yes | No | No |  |
| Lee et al. (2015) | Yes | Yes | Yes | Yes | No | No |  |
| Prabhakaran & Wei (2019) | Yes | Yes | Yes | No | No | Yes |  |
| Ringbæk et al. (2015) | Yes | Yes | Yes | Yes | Yes | No |  |
| Soriano et al. (2018) | Yes | Yes | Yes | Yes | Yes | No |  |
| Tupper et al. (2018) | Yes | Yes | Yes | Yes | Yes | No |  |
| **4. Diabetes** | | | | | | |  |
| Fernandes et al. (2016) | Yes | Yes | Yes | Yes | No | No |  |
| Hansen et al. (2017) | Yes | Yes | Yes | Yes | Yes | No |  |
| Hemmati Maslakpak et al. (2017) | Yes | Yes | Yes | No | No | No |  |
| Kim & Utz (2019) | Yes | Yes | Yes | Yes | No | No |  |
| Kim et al. (2021) | Yes | Yes | Yes | Yes | Yes | No |  |
| Li et al. (2017) | Yes | Yes | Yes | Yes | No | Yes |  |
| Odnoletkova et al. (2016) | Yes | Yes | Yes | Yes | No | No |  |
| Sherifali et al. (2021) | Yes | Yes | Yes | No | No | Yes |  |
| Tan et al. (2020) | Yes | Yes | Yes | No | No | Yes |  |
| **5. Cancer** | | | | | | |  |
| Beaver et al. (2017) | Yes | Yes | Yes | Yes | No | No |  |
| Çınar et al. (2021) | Yes | Yes | Yes | Yes | Yes | No |  |
| Ghanbari et al. (2021) | Yes | Yes | Yes | No | No | No |  |
| Wheelock et al. (2015) | Yes | Yes | Yes | Yes | Yes | Yes |  |
| **6. Stroke** | | | | | | |  |
| Irewall et al. (2015)  Irewall et al. (2019)  Ögren et al. (2015) | Yes | Yes | Yes | Yes | No | No |  |
| Kirkness et al. (2017) | Yes | Yes | Yes | No | No | No |  |
| Wan et al. (2016) | Yes | Yes | Yes | Yes | No | No |  |
